# Early genome erosion and internal phage-symbiont-host interaction in the endosymbionts of a cold-seep tubeworm

**DOI:** 10.1016/j.isci.2023.107033

**Published:** 2023-06-07

**Authors:** Zhao-Ming Gao, Ting Xu, Hua-Guan Chen, Rui Lu, Jun Tao, Hong-Bin Wang, Jian-Wen Qiu, Yong Wang

**Affiliations:** 1 Institute of Deep Sea Science and Engineering, Chinese Academy of Sciences, Sanya 572000, China; 2 HKUST-CAS Sanya Joint Laboratory of Marine Science Research, Chinese Academy of Sciences, Sanya 572000, China; 3 Department of Ocean Science, The Hong Kong University of Science and Technology, Hong Kong, China; 4 Southern Marine Science and Engineering Guangdong Laboratory (Guangzhou), Guangzhou 511458, China; 5 University of Chinese Academy of Sciences, Beijing 101408, China; 6 MLR Key Laboratory of Marine Mineral Resources, Guangzhou Marine Geological Survey, China Geological Survey, Guangzhou 511458, China; 7 Department of Biology, Hong Kong Baptist University, Hong Kong, China; 8 Institute for Ocean Engineering, Shenzhen International Graduate School, Tsinghua University, Shenzhen 518000, China

**Keywords:** Zoology, Molecular biology, Evolutionary biology

## Abstract

Endosymbiosis with chemosynthetic Gammaproteobacteria is widely recognized as an adaptive mechanism of siboglinid tubeworms, yet evolution of these endosymbionts and their driving forces remain elusive. Here, we report a finished endosymbiont genome (HMS1) of the cold-seep tubeworm *Sclerolinum annulatum*. The HMS1 genome is small in size, with abundant prophages and transposable elements but lacking gene sets coding for denitrification, hydrogen oxidization, oxidative phosphorylation, vitamin biosynthesis, cell pH and/or sodium homeostasis, environmental sensing, and motility, indicative of early genome erosion and adaptive evolution toward obligate endosymbiosis. Unexpectedly, a prophage embedded in the HMS1 genome undergoes lytic cycle. Highly expressed ROS scavenger and LexA repressor genes indicate that the tubeworm host likely activates the lysogenic phage into lytic cycle through the SOS response to regulate endosymbiont population and harvest nutrients. Our findings indicate progressive evolution of *Sclerolinum* endosymbionts toward obligate endosymbiosis and expand the knowledge about phage-symbiont-host interaction in deep-sea tubeworms.

## Highlights

• A finished endosymbiont genome of *Sclerolinum* tubeworm was assembled
• It shows signs of early genome erosion and obligate endosymbiotic lifestyle
• The symbiont evolution was mediated by mobile genetic elements
• An active phage likely has interactions with the symbiont and the host

## Graphical abstract

**Figure:**
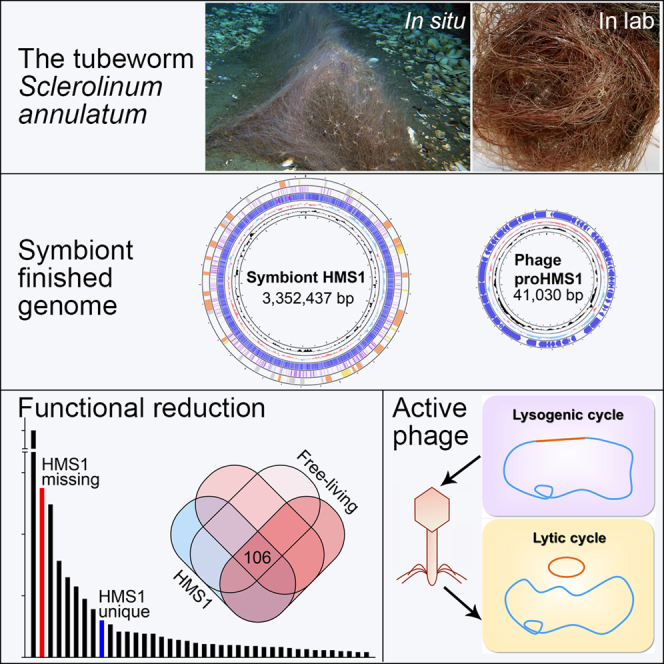

## Introduction

Due to the limited availability of organic matter from the ocean surface, the majority of the deep-sea floor is characterized by low biomass.^1^ However, at hydrothermal vents, cold seeps and organic falls, several groups of invertebrates including siboglinid tubeworms, vesicomyid clams, bathymodiolin mussels, and peltospirid snails have formed symbiotic relationships with chemosynthetic bacteria that use reducing substances such as hydrogen sulfide, methane and hydrogen.^2^^,^^3^ Such chemosymbiosis provides the hosts with most or even all of their energy and nutrition, allowing these specialized deep-sea invertebrates to develop into dense populations that rival the intertidal mussel beds in biomass.^4^ Studies over the last four decades have provided insights into the phylogenetic positions of these specialized invertebrates, and the molecular mechanisms of symbiosis between these invertebrates and their chemosynthetic bacteria.^3^^,^^5^

It is widely known that deep-sea tubeworm species in the family Siboglinidae (Annelida) usually depend on sulfur-oxidizing bacteria for carbon fixation by using reductive fluids as an energy source.^2^ Siboglinidae consists of four main lineages, including the giant tubeworms Vestimentifera, the bone-eating *Osedax*, and the slender tubeworms *Sclerolinum* and Frenulata.^6^ Among them, vestimentiferans inhabiting hydrothermal vents and cold seeps are conspicuous and structure-forming, provide a habitat for many other invertebrates, and thus have been extensively studied.^7^^,^^8^^,^^9^ Because vestimentiferans obtain nutrition from their endosymbionts, they lack both the mouth and gut. Vestimentiferans usually host a single ribotype of sulfur-oxidizing Gammaproteobacteria,^10^ although polyclonal endosymbionts have also been reported.^11^^,^^12^ Vestimentiferan endosymbionts are horizontally acquired from free-living pools during the host’s larval stage and maintained in a specialized organ of the adult termed trophosome.^13^ When the host dies, the endosymbionts are released back into the ambient environment.^14^ Nevertheless, no information is available on the transmission mode of the *Sclerolinum*, *Osedax*, and Frenulata symbionts.

Siboglinid endosymbionts are currently unculturable. Their host/geographic specificity, genetic diversity, metabolic potentials and symbiotic mechanisms have only been investigated by culture-independent molecular methods. Metagenomic analyses have been used to assemble more than ten siboglinid endosymbiont genomes.^10^^,^^15^^,^^16^^,^^17^^,^^18^^,^^19^^,^^20^^,^^21^^,^^22^ Analyses of the known Vestimentiferan and Frenulata (the *Galathealinum* genus) gammaproteobacterial endosymbionts indicate that they likely employ mixotrophic lifestyles and have conserved mechanisms for host infection.^16^^,^^17^ In contrast, endosymbionts of the distantly related bone-eating *Osedax* tubeworms belong to the order Oceanospirillales and appear to be heterotrophic.^23^ Nevertheless, because of a lack of sequencing effort on *Sclerolinum* species, the metabolic potential and genetic diversity of their symbionts remain unknown.

**Figure 1:**
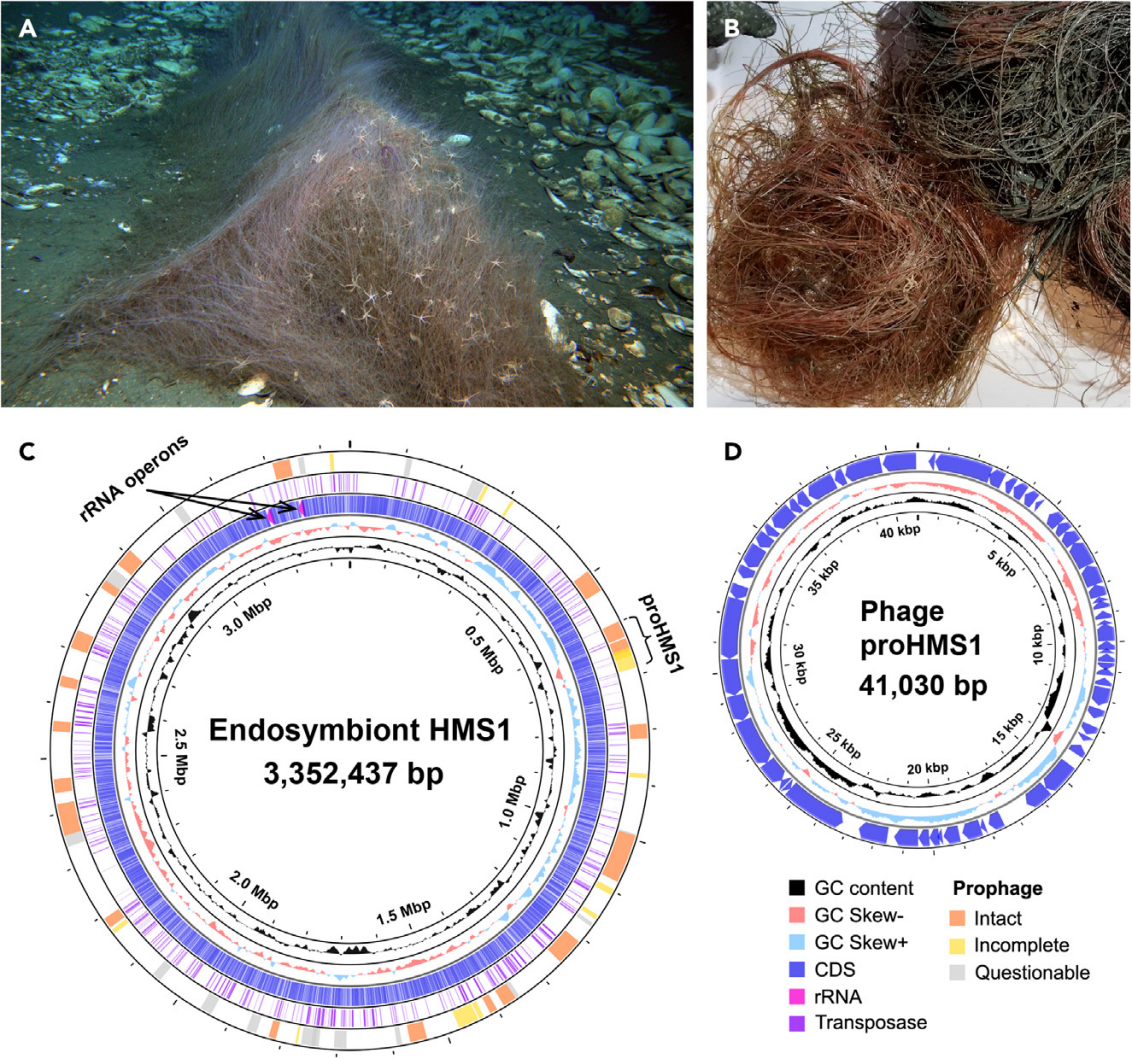
Photography of the tubeworm *Sclerolinumannulatum* and genomic view of its endosymbiont HMS1 and phage proHMS1 (A) *In situ* photograph of a population of *S*. *annulatum* on the Haima cold seep. (B) Its specimens on aboard. (C) Key features of the circular HMS1 genome. (D) Key features of the circular proHMS1 genome. For both HMS1 and proHMS1, their genome size was indicated in the center. From the innermost to the outermost, the first circle represents GC content, the second circle represents GC skew, the third circle shows open reading frames (CDS, tRNA, and rRNA), the fourth circle shows genes encoding transposases, and the fifth circle shows genomic regions of prophages predicated using the PHAST web server. Details of the intact, incomplete and questionable prophages are in Table S13. Locations of the rRNA operons and proHMS1 are labeled on the HMS1 genome. Genomic features were plotted with CGView Server (http://cgview.ca/). Abbreviation: CDS, coding sequence.

The genus *Sclerolinum* is the sister clade to vestimentiferans. They have been reported from deep-sea cold seep sediments and wood falls.^24^^,^^25^ Among the eight species of *Sclerolinum*, only *Sclerolinum contortum* has been examined for its endosymbionts through light and electron microscopy, bacterial 16S rRNA gene amplification and fluorescent *in situ* hybridization,^26^^,^^27^^,^^28^ yet no metagenomic studies have been carried out for them. The recent discovery of *Sclerolinum annulatum* (Figures 1A and 1B) from the Haima cold seep in the South China Sea has provided an opportunity to fill this knowledge gap.^29^ Here, we employed culture-independent metagenomic and metatranscriptomic approaches to characterize the endosymbionts of *S*. *annulatum*. We successfully obtained a complete genome of the endosymbiont. For the first time, we found remarkable functional reduction but abundant mobile genetic elements in a siboglinid endosymbiont indicative of the early stage of genome erosion and a host-restricted lifestyle. Unexpectedly, we found that a prophage embedded in the endosymbiont genome was activated to enter the lytic cycle and may help the tubeworm host to regulate the population size of the endosymbionts.

## Results

### Endosymbiont genome recovery and metatranscriptomes

Specimens of *S*. *annulatum* were collected from the Haima cold seep at 1,425 m depth in May, 2019 (Figures 1A and 1B). The tubeworms formed dense aggregations with empty shells of the vesicomyid clams scattered around (Figure 1A). A sediment core collected adjacent to the tubeworm colony was rich in hydrogen sulfide and gas hydrate. Using both Illumina and PacBio sequencing reads from one tubeworm, we successfully assembled a complete circular bacterial genome (hereafter referred to as HMS1) with a total length of 3.35 Mbp and a GC content of 50.41% (Table 1, Figure 1C, and Table S1). HMS1 harbors two rRNA operons with their 16S rRNA genes having two base mismatches (Figures 1C and S1). The two 16S rRNA genes entirely match the previously reported OTU-1 phylotypes that represent the endosymbiont of *S*. *annulatum*.^29^ Phylogenetic analysis of the 16S rRNA gene sequences (1,495 bp) consistently showed high similarities between HMS1 and the endosymbionts hosted by *S*. *contortum* from the Gulf of Mexico (GenBank: HE614013) and the Haakon Mosby Mud Volcano (GenBank: AM883183) with > 99.6% sequence identity (Figure S2). These results indicate that HMS1 represents the endosymbiont of *S*. *annulatum*.^29^

**Table 1:** General genomic features of the endosymbiont HMS1 and its relatives

Genome^a^	Genome size (Mbp)	N_50_ (Kbp)	No. of contigs	% GC	No. of CDSs	No. of signal peptides	% coding density	Host
HMS1	3.35	NA	1	50.41	4,129	130	80.80	*S*. *annulatum*
MCR1	4.26	89.9	82	50.52	3,886	349	88.14	MCR vestimentiferans
MCR2	4.64	42.8	200	50.19	4,205	373	86.18	
*Paraescarpia echinospica*	4.06	381.7	14	53.5	3,522	366	87.28	Seep vestimentiferans
*Seepiophila jonesi*	3.53	20.7	323	54.39	3,561	312	86.05	
*Lamellibrachia luymesi*	3.53	20.6	337	54.36	3,559	311	85.84	
*Lamellibrachia barhami*	4.17	4.15	19	53.94	3,884	366	85.88	
*Escarpia laminata*	4.25	124.9	71	54.52	3,768	385	88.73	
*Escarpia piscesae*	4.06	313.6	23	54.18	3,700	395	89.62	
*Riftia pachyptila*	3.48	29.7	197	58.82	3,364	290	87.10	Vent vestimentiferans
*Ridgeia piscesae*	3.44	84.0	97	58.88	3,133	316	88.87	
*Tevnia jerichonana*	3.64	92.7	184	58.16	3,312	308	86.34	
*Galathealinum brachiosum*	3.77	726.6	14	38.89	3,450	404	88.66	Frenulata
*Sedimenticola thiotaurini*	3.96	NA	1	56.83	3,612	302	88.41	Free-living
*Sedimenticola selenatireducens*	4.56	223.5	41	56.65	4,240	384	87.88	
*Sedimenticola endophacoides*	2.98	27.7	172	63.86	3,421	197	87.36	Lucinidae

a The siboglinid symbionts were labeled using the names of their hosts. Data for HMS1 is from this study. Data for the relatives come from our annotations of their genomes. Detailed information is shown in Tables S1 and S2. Abbreviations: CDS, coding sequence; NA, not applicable.

**Figure 2:**
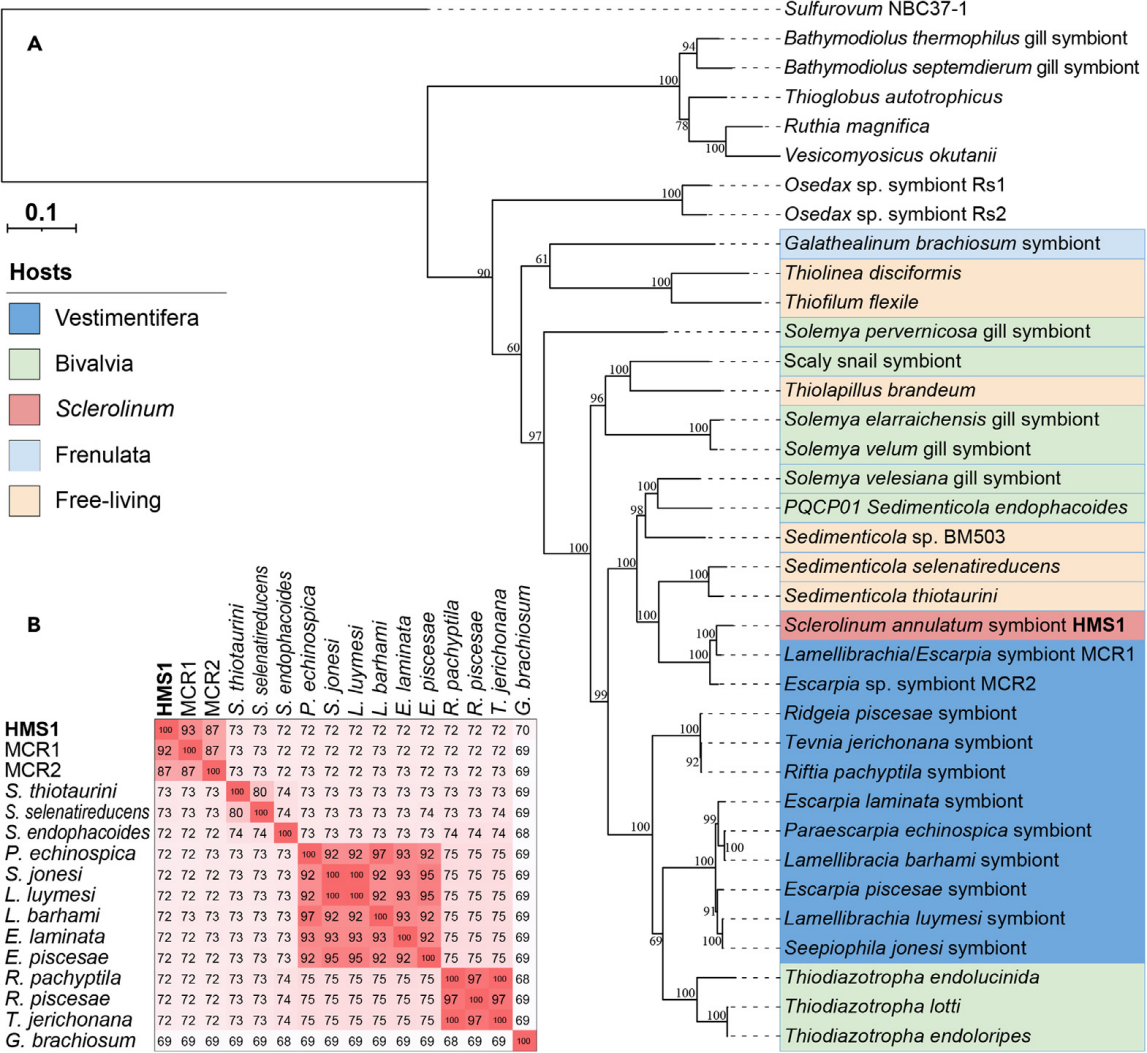
Phylogenomic analysis and ANI calculations for the endosymbiont HMS1 and its relatives (A) Maximum-likelihood tree of tubeworm endosymbionts based on 43 concatenated conserved proteins derived from CheckM was constructed with the PROTGAMMALG model using raxmlGUI v1.5 for 100 replicates. Bootstrap values are shown as percentages on the branches. The scale bar indicates the mean number of amino acid substitutions per site. *Sulfurovum* sp. NBC37-1 was used as the outgroup. The GenBank accession number of genomes present in the tree and respective references are provided in Table S2. (B) The ANI values between HMS1 and its relatives. A full version of ANI calculations can be found in Table S3.

Phylogenomic analysis based on 43 conserved bacterial proteins revealed that HMS1 formed a well-supported clade with the endosymbionts of *Escarpia* sp. and *Lamellibrachia* sp. 2 (the MCR1 and MCR2 symbionts, Figure 2A) from a low-temperature hydrothermal diffuse vent along the Mid-Cayman Rise (MCR).^16^ HMS1 is also closely related to the free-living *Sedimenticola* species collected from estuarine or salt marsh sediment, the gill symbionts hosted by the mangrove lucinid *Phacoides pectinatus*^30^ and the intertidal mudflat solemyid *Solemya velesiana*,^31^ whereas it is distant from other vestimentiferan endosymbionts from vents and seeps (Figure 2A and Table S2).^21^ HMS1 is even more distantly related to the *Galathealinum* endosymbiont.^17^ Although HMS1 is closely related to the MCR endosymbionts in the 16S rRNA gene (98.5–99.1% identity) and in the phylogenomic tree (Figure 2A), and is highly similar in GC content (50.41% vs. 50.19–50.52%, Table 1), genome rearrangement analysis indicates that the HMS1 genome is structurally highly distinct from the MCR genomes (Figure S3). Furthermore, the average nucleotide identity (ANI) values between the HMS1 and MCR genomes are < 92.5%, lower than the intraspecific ANI threshold of 95% (Figure 2B and Table S3).^32^ The ANI values between HMS1 and the other reference genomes are even lower (Figure 2B and Table S3). Therefore, HMS1 should be a novel species in the same genus as the MCR endosymbionts that is *Candidatus* Vondammii,^16^ and herein named as *Candidatus* Vondammii deminuti (*deminuti*: decreasing in Latin, referring to early genome reduction).

Three individuals of *S*. *annulatum* stored in RNAlater at −80°C were used for transcriptomic analysis. Metatranscriptomic sequencing produced 54 million, 109 million, and 105 million clean paired-end reads, respectively (Table S4). Among them, a total of 1,844,893, 1,250,911, and 2,780,863 reads were mapped to the coding sequences (CDSs) of HMS1, respectively. Of the total 4,129 CDSs, 3,782 CDSs (91.6%) mapped by metatranscriptomic reads of at least two of the three individuals were considered expressed (Figure S4 and Table S5). Average transcripts per million value (TPM_av_) of the expressed genes varied from 0.21 to 26114.27, with the top 10% highly expressed genes having a TPM_av_ of > 540. Gene expression levels among the biological replicates were highly correlated (Pearson correlation coefficients ranging from 0.73 to 0.91, Figure S4), indicating their high transcriptional consistency.^33^ Metatranscriptomic analysis revealed that two *iscR* genes involved in the transcription of iron-sulfur cluster assembly and a number of housekeeping genes encoding ribosomal proteins and chaperonins are the most actively expressed genes. Notably, the genes encoding antioxidant enzymes including a superoxide dismutase and a peroxiredoxin, as well as the *lexA* and *recA* genes that take part in the SOS response, also ranked among the actively expressed genes.^34^

### Reduced genome of the endosymbiont HMS1

In light of the phylogenetic inference (Figure 2A), we structurally and functionally compared HMS1 with those of the vestimentiferan endosymbionts and the representative *Sedimenticola* species (the free-living *Sedimenticola selenatireducens* and *Sedimenticola thiotaurini*, and the symbiotic *Sedimenticola endophacoides* of the lucinid *P*. *pectinatus*) (Table 1). As a far distinct relative, the *Galathealinum* endosymbiont was excluded from the comparison. The *S*. *velesiana* gill symbiont was also excluded because of the low completeness (86.18%) of its genome assembly (Table S6).

**Figure 3:**
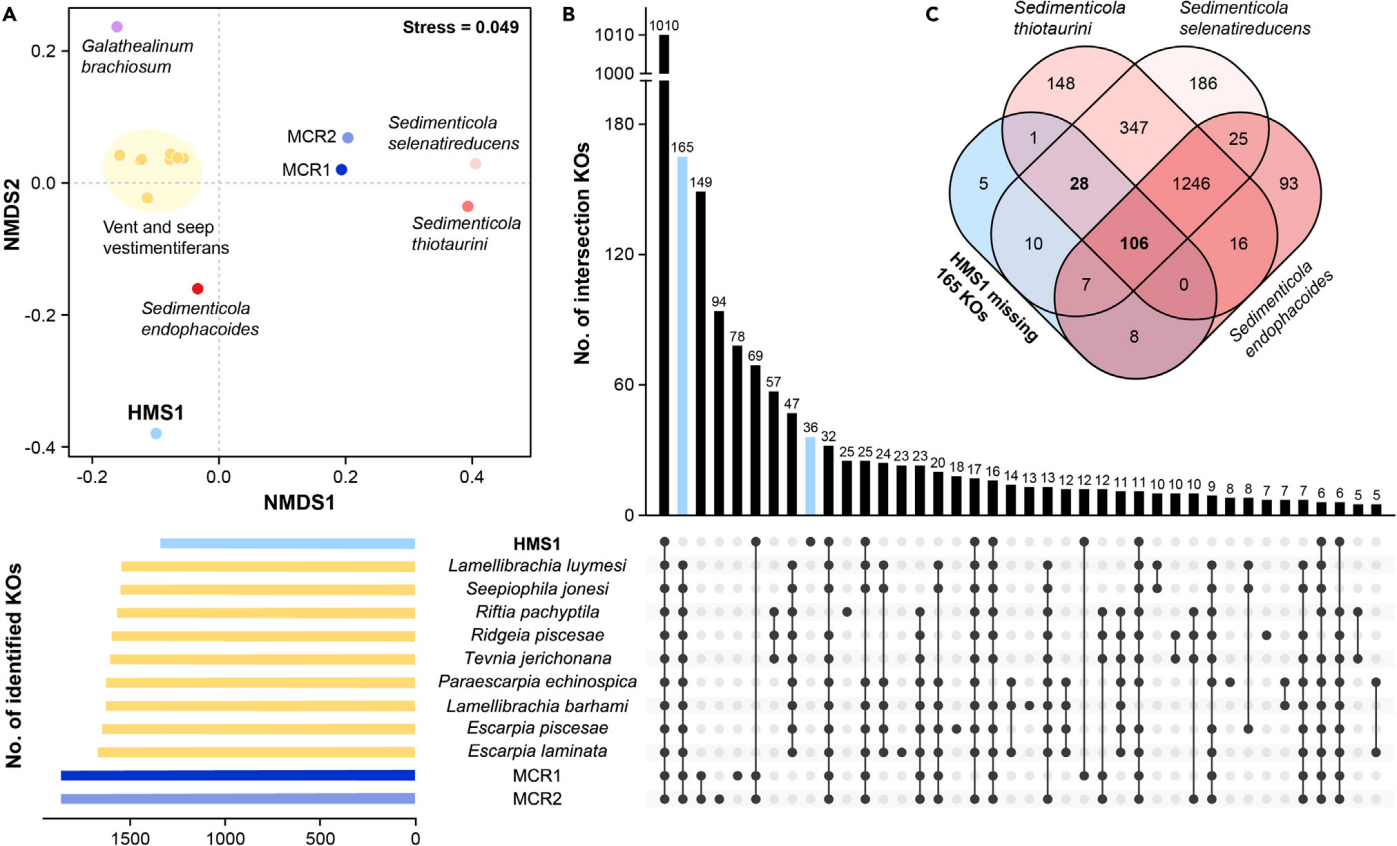
Functional comparison of the endosymbiont HMS1 and its relatives (A) Non-metric multidimensional scaling (nMDS) ordination of HMS1 and reference genomes based on KEGG annotations. The two-dimensional stress value for the nMDS was 0.049 based on the Bray-Curtis distance. Analysis was performed using PRIMER-E based on the relative abundance of KOs. (B) UpSet diagram of unique and shared KOs for HMS1 and the vestimentiferan endosymbionts. (C) Venn diagram shows the number of intersected KOs between the 165 HMS1-missing KOs and the *Sedimenticola* species. Upset and Venn diagrams were drawn using the BioLadder cloud server (https://www.bioladder.cn/).

The HMS1 genome is smaller (3.35 Mbp) than those of the closely related MCR endosymbionts (4.26 Mbp and 4.64 Mbp) and the free-living *Sedimenticola* species (3.99 Mbp and 4.59 Mbp), but larger than the symbiotic *S*. *endophacoides* genome (2.98 Mbp) (Table 1). The HMS1 genome is also smaller than those of the other vent and seep siboglinid endosymbionts (3.44 Mbp to 4.06 Mbp). Meanwhile, HMS1 has a much lower number of signal peptide-containing genes (n = 130) compared to the vestimentiferan endosymbionts (n = 290–404), the free-living *Sedimenticola* species (n = 302–384) and the symbiotic *S*. *endophacoides* (n = 197) (Table 1). In addition, the HMS1 genome has a lower coding density (80.80%) than all the compared genomes (85.84–89.62%). However, HMS1 has more CDSs (n = 4,129) than most of the vestimentiferan endosymbionts (n = 3133–4180) and *Sedimenticola* species (n = 3,421–4,240) (Table 1), mainly due to more transposase genes in HMS1 than in the compared genomes (1,057 vs. 8–223). When transposase genes are excluded, HMS1 has fewer CDSs than all the compared genomes (Table S1). HMS1 also has the smallest number of CDSs assigned to KEGG Orthologies (KOs) and COG categories after excluding transposase genes (Table S1). Functional clustering based on KEGG/COG/PFAM annotations showed that HMS1 is clearly separated from the vestimentiferan endosymbionts and the free-living *Sedimenticola* species (Figures 3A and S5). Instead, as phylogenetically close relatives of HMS1, the MCR endosymbionts exhibit a much closer relationship with the free-living *Sedimenticola* species and vestimentiferan endosymbionts than with HMS1 (Figure 3A).

**Figure 4:**
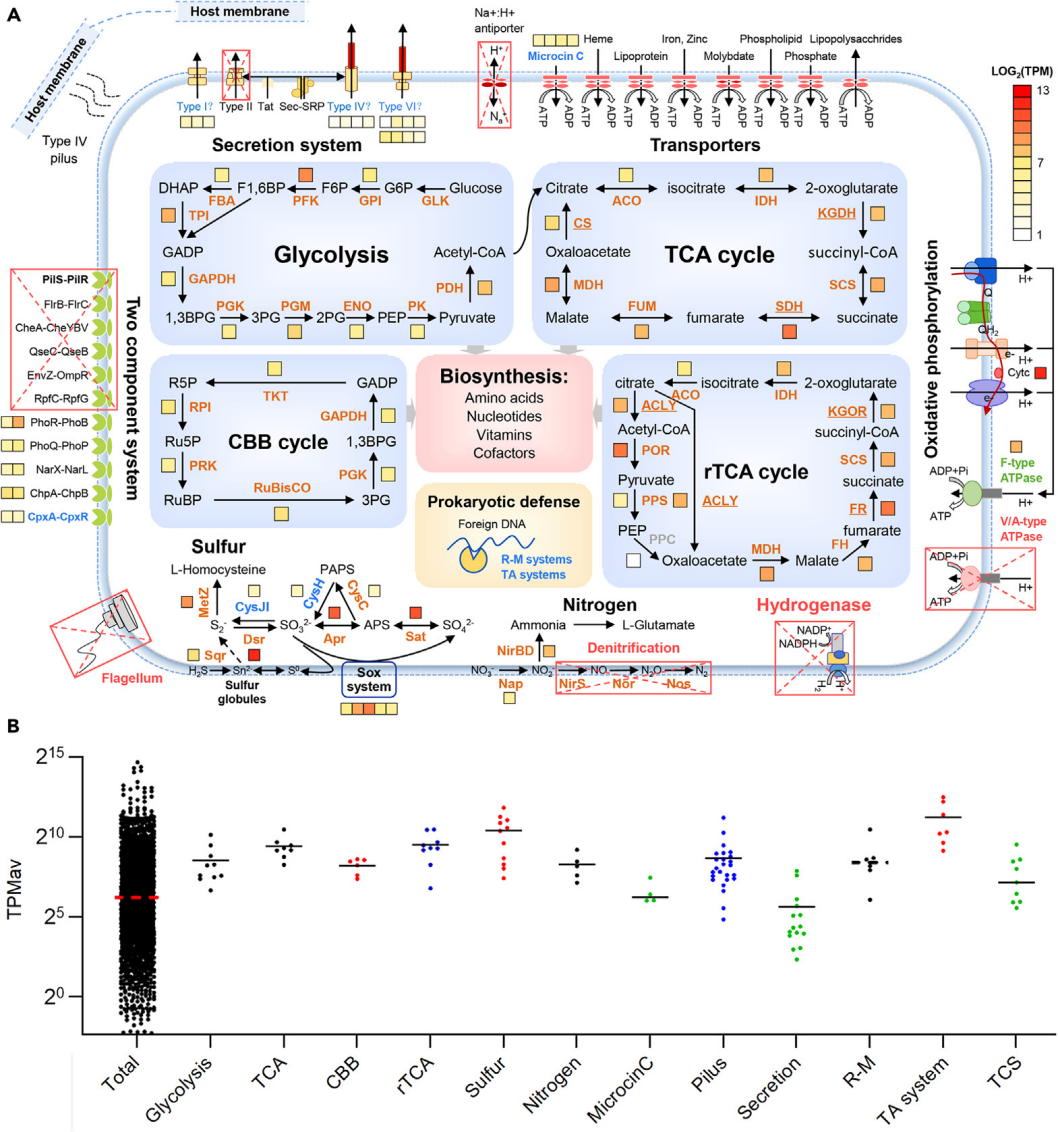
Schematic overview of the lifestyle of the *Sclerolinumannulatum* endosymbiont HMS1 (A) Major cellular features and central metabolism pathways. Boxes adjacent to genes are colored according to the log-transformed TPM_av_ values (mean of three replicates) to represent expression levels. Functions missing in HMS1 are indicated by red rectangles with a cross. Functions present in HMS1 but absent in most or all other tubeworm endosymbionts are highlighted in blue. (B) Expression of selected KEGG pathways and Brites. The median expression level in each group is represented by a solid line.

UpSet diagram further illustrates the divergence between HMS1 and the vestimentiferan endosymbionts (Figure 3B). Among the 2,421 KOs identified in the analyzed genomes, 1,010 are present in all genomes. HMS1 has 36 unique KOs but lacks 165 KOs that are present in all vestimentiferan endosymbionts. Of interest, 134 of the 165 HMS1-missing KOs are present in the two free-living *Sedimenticola* species and 106 are even present in the symbiotic *S*. *endophacoides* (Figure 3C). Functional interpretation shows that the HMS1-missing KOs include genes with important functions, such as energy and nutrient metabolisms, cell pH and/or sodium homeostasis, environmental sensing, motility, and secretion (Figures 4 and S6). HMS1 lacks extra 149 KOs when only compared to its two closely relative MCR endosymbiont genomes (Figure 3B).

### Lost functions of the endosymbiont HMS1

#### Energy and nutrient metabolisms

The genes involved in key energy and nutrient metabolisms are summarized in Figure 4A and Table S7. HMS1 has all the genes in the Calvin–Benson–Bassham (CBB) cycle and the reductive tricarboxylic acid (rTCA) cycle for carbon fixation, as well as genes encoding the *sox* system and the reverse sulfate reduction pathway for sulfur oxidization, confirming the conservation of these core metabolic pathways across siboglinid chemoautotrophic symbionts.^17^^,^^20^ Genes involved in the rTCA cycle were expressed at a higher level than those in the CBB cycle in HMS1 (Figure 4B), which is consistent with the findings of vestimentiferan endosymbionts.^15^^,^^20^ However, the group I NiFe type hydrogenases (HyaBA: KEGG KOs K06281 and K06282) that are present in all the vestimentiferan endosymbionts and *Sedimenticola* species are missing in HMS1. HMS1 retains the *napABC* and *nirBD* genes that constitute the intact dissimilatory nitrate reduction pathway, but lacks dissimilatory denitrifying genes (*nirS*, *norBC* and *nosZ*) that are present in all the vestimentiferan endosymbionts and *Sedimenticola* species. All the available vestimentiferan endosymbiont genomes encode both the F- and V-type ATPases, but HMS1 lacks a whole set of *atpABCDEIK* genes encoding the V-type ATPase. In addition, HMS1 lacks the *men* and *cob* genes for menaquinone (vitamin K2) and cobalamin (B12) synthesis. The loss of these genes indicates a simpler metabolic capability of the *S*. *annulatum* endosymbiont.

#### Transporters

HMS1 has 119 genes assigned to transporters (KEGG Brite: ko02000), substantially fewer than the number of such genes in the vestimentiferan endosymbionts (n = 154–200), the free-living *Sedimenticola* species (n = 202–218) and the symbiotic *S*. *endophacoides* (n = 146) (Table S8). The genes encoding iron (III) transporter (*afuABC*) and peptides/nickel transporter (ABC.PE.E, ABC.PE.E, ABC.PE.A) that are present in all the vestimentiferan endosymbionts and free-living *Sedimenticola* species are missing in HMS1. Notably, the *nhaA* and *mnhBCDEFG* genes encoding Na^+^/H^+^ antiporters that function in cellular salt and pH homeostasis^35^ are present in the vestimentiferan endosymbionts and *Sedimenticola* species but absent in HMS1 (Figure 4A).

#### Two-component systems (TCSs)

The TCSs genes are summarized in Table S9. HMS1 encodes 29 genes assigned to TCSs (KEGG Brite: ko02022), which is fewer than the number of such genes in the vestimentiferan endosymbionts (n = 46–61), the free-living *Sedimenticola* species (n = 59–60), and the symbiotic *S*. *endophacoides* (n = 42). The PhoR-PhoB and PhoQ-PhoP TCSs that have been proposed to mainly sense host-related signals and mediate adaptation of symbionts to the host’s internal environment^36^^,^^37^ are present in all the analyzed tubeworm endosymbionts including HMS1, and the respective CDSs of HMS1 are expressed (Figure 4B), indicating their essential role in maintaining the endosymbiosis. Unlike the vestimentiferan endosymbionts and free-living *Sedimenticola* species, HMS1 has lost genes encoding the QseC-QseB, EnvZ-OmpR, PilS-PilR, CheA-CheYBV, RpfC-RpfG and FlrB-FlrC TCS systems (Figure 4A). These TCS modules, which function in bacteria’s free-living stage, are responsible for regulating cell motility and biofilm formation through quorum sensing, and are required for the transcription of genes responsible for bacterial adherence and virulence.^38^^,^^39^^,^^40^^,^^41^^,^^42^^,^^43^ The loss of these TCSs indicates a reduced capability of the *S*. *annulatum* endosymbiont for environmental sensing.

#### Motility capability

All the available vestimentiferan endosymbiont genomes harbor the genes encoding flagella and T4P motility machinery as well as chemotaxis proteins (Figure 4A and Table S10). In contrast, HMS1 has lost all genes encoding the flagella and chemotaxis proteins (Table S10). As a two-component system, PilS-PilR has been known to regulate the expression of the T4P major subunit PilA in *Pseudomonas aeruginosa*, and the T4P phenotype is missing in a *pilR* mutant.^44^ HMS1 loses the *pilS* and *pilR* genes and thus likely has an incomplete T4P system. The lack of the flagella machinery and chemotaxis protein coupled with the incomplete T4P system all indicate a restricted motility capability of the *S*. *annulatum* endosymbiont.

#### Secretion systems

Type II secretion system (T2SS) has been suggested to export hemolysins and chitinases for the siboglinid endosymbionts to penetrate the host cells and migrate to the newly developed trophosome.^17^ A complete set of genes encoding T2SS system are present in the vestimentiferan endosymbionts and *Sedimenticola* species but missing in HMS1. Furthermore, gene sets encoding the T1SS, T4SS and T6SS systems for macromolecule export are either incomplete in HMS1 compared to vestimentiferan endosymbionts or lowly expressed (Figure 4A, Note S1, and Table S11).

### Highly abundant and actively expressed mobile genetic elements

The HMS1 chromosome architecture is complex with highly abundant mobile genetic elements (MGEs), including prophages and transposable elements (TEs) (Figure 1C). A total of 71 genes encoding phage proteins were identified in HMS1 according to the COG annotation, which is much more abundant than 4–20 such genes in the vestimentiferan endosymbionts and 5–10 such genes in the *Sedimenticola* species (Table S12). Searching against the PHASTER web server^45^ further revealed 47 prophage regions in HMS1, of which 19 regions are intact (Figure 1C and Table S13). These prophages, ranging from 4.0 to 88.8 kb in size, add up to 1.03 Mbp and account for 30.7% of the HMS1 genome. The density of prophage elements in the HMS1 genome is close to that of the insect parasite *Arsenophonus nasoniae*, whose genome encodes 55 prophage regions — the highest number of prophage complements reported to date.^46^ Alignment view using the ViPTree online server^47^ shows that the prophage elements 4, 6, 43 and 44 have syntenic regions that may come from the same ancestral phage through multiple invasions or transpositions (Figure S7). Several HMS1 genes belonging to the phage integrase family show high expression with TPM_av_ values up to 437.36 (Table S14).

HMS1 encodes 1,057 transposase genes as revealed by the COG annotation, compared to 8–223 and 20–49 such genes in the vestimentiferan endosymbionts and *Sedimenticola* species, respectively (Table S15), suggesting an extensive expansion of TEs in the *S*. *annulatum* endosymbiont. These transposase genes are located not just in the prophage regions, but relatively evenly distributed on the genome except for several regions having no insertions (Figure 1C). Annotation of the transposases in HMS1 using the ISfinder database and categorization by insertion sequence (IS) family^48^ further revealed 986 transposase genes in 895 ISs (Table S16). Among them, 716 transposase genes are putatively truncated, while 201 transposase genes are complete and probably functional. Metatranscriptomic analysis showed that a large number of transposase genes were expressed, with some among the top 10% highly expressed genes (Table S17).

### A prophage undergoing lytic cycle

Unlike the simple holobiont system between vestimentiferan tubeworms and their endosymbionts, the *S*. *annulatum* holobiont appears to be more complex with the participation of an active prophage here termed proHMS1 (Figure 1D). PacBio long reads mapping showed that proHMS1 could be integrated into the endosymbiont genome or be a circular genome, suggesting that it has both lysogenic and lytic lifestyles (Figure S8). The extra higher reads coverage on the phage region compared to the endosymbiont genome also indicates the lytic activity of proHMS1. The annular phage genome has a total length of 41,030 bp (Figure 1D). Taxonomic classification by ViPTree^47^ showed that proHMS1 is affiliated with the family Siphoviridae (Figure S9). The proteomic tree also showed that proHMS1 is far distant from other previously known phages (Figure S9). Genes involved in phage core functions such as head, tail, capsid, terminase, recombinase and lysozyme were annotated, of which most were expressed, yet no auxiliary metabolic genes (AMGs) that modulate host cell metabolism were found (Table S18). Analyzing the metagenomic data from ten *S*. *annulatum* individuals shows that proHMS1 is highly abundant in nine of them, but almost undetectable in the individual HM_W20 (Table S19). Correspondingly, the mean coverage of the endosymbiont HMS1 normalized by 100X mitochondrial sequence is 54.19X in HM_W20, higher than the values in eight other individuals (5.27–39.33X), which suggests a relatively higher abundance of the endosymbionts in proHMS1-missing tubeworm individuals.

### Anti-viral defense systems

Most of the compared siboglinid endosymbionts and the two free-living *Sedimenticola* species have Clustered Regularly Interspaced Short Palindromic Repeats (CRISPR) spacers and encode CRISPR-Associated (Cas) proteins, but HMS1 lacks CRISRP arrays (Table S1, annotated through the Prokka v1.11 program) and Cas proteins (Table S20). CrisprMiner2^49^ further confirms the missing of CRISPR-Cas system in HMS1. Instead, HMS1 has a number of genes encoding types I and II restriction-methylation (R-M) defense systems and types II (YoeB-YefM, HigB-HigA and VapC-VapB) and III (CptA-CptB) toxin-antitoxin (TA) systems (Table S20). Metatranscriptomic analysis further shows that most of the R-M and TA genes were expressed in HMS1 and some of them fell into the top 10% highly expressed genes with TPM_av_ up to 5781.71 (Tables S5 and S20). In addition, a gene encoding DNA methyltransferase HhaI in the proHMS1 genome was expressed with an extremely high TPM_av_ of 1692.34 (Table S18).

## Discussion

The rapid development of sequencing technologies has made it possible to produce high-quality genomes of tubeworm endosymbionts recently. Before this study, a highly contiguous siboglinid endosymbiont genome from *Lamellibrachia barhami*^21^ and a complete genome from *Riftia pachyptila* were available.^22^ In the present study, we successfully assembled a complete genome of the endosymbiont *Ca*. Vondammii deminuti (HMS1) from *S*. *annulatum*, which represents the first endosymbiont genome of *Sclerolinum* tubeworms. Our identification of HMS1 as a novel species in the genus *Candidatus* Vondammii^16^ expands the genetic diversity of tubeworm endosymbionts and provides a useful reference for further systemic analyses of chemosynthetic bacteria.

Symbiosis in prokaryotes is a progressive process that could lead to genome reduction and functional deficiency.^50^ Vertically transmitted symbionts of marine animals are apt to have a small genome and only retain essential functions that are beneficial to their hosts.^51^^,^^52^^,^^53^ However, the currently known siboglinid endosymbionts have a genome size of up to 4.64 Mbp, without obvious reduction when compared with their relatives living in natural environments.^16^ Such endosymbionts have functional traits that are necessary for their survival during the free-living stage.^10^^,^^18^^,^^22^ In comparison with the vestimentiferan endosymbionts and the free-living *Sedimenticola* species, HMS1 has undergone remarkable genome erosion. HMS1 has a smaller genome size compared to other tubeworm endosymbionts, lower coding density, a smaller number of CDSs and functional genes (when excluding transposase genes), and fewer signal-peptide-containing genes. All of these genome-level findings imply that the *S*. *annulatum* endosymbiont has likely undergone reductive evolution toward obligate endosymbiosis without a free-living stage,^50^^,^^54^ yet direct evidences for their vertical transmission lifestyle are yet unavailable.

Motility machines are typical traits to distinguish horizontally and vertically transmitted symbionts. For instance, the horizontally transmitted bathymodiolin symbionts and mixed-mode transmitted solemyid symbionts have genes encoding motility machines, yet the vertically transmitted vesicomyid symbionts have lost them.^31^^,^^53^^,^^55^ The siboglinid tubeworms have been inferred to horizontally acquire their endosymbionts from the ambient environments.^14^ Flagella and type IV pilus (T4P) and associated chemotaxis proteins that are essential for tubeworm symbionts to approach their hosts^17^ and attach to the host surface^56^ are encoded by their genomes. The loss of these genes in HMS1 implies that the *S*. *annulatum* endosymbiont is unable to reach tubeworm hosts like their free-living relatives. TCSs are used by bacteria to sense and respond to diverse environmental stresses and host signals.^57^ The lack of those TCSs in HMS1 for quorum sensing, adherence and virulence of free-living bacteria indicates a reduced capability of the *S*. *annulatum* endosymbiont for environmental sensing. Na^+^/H^+^ antiporters in prokaryotic and eukaryotic membranes involve in the regulation of salt and pH homeostasis.^35^ Tubeworm endosymbionts that are acquired from the external environments may confront various environmental challenges during their free-living stage, which necessitates the use of Na^+^/H^+^ antiporters to maintain cell homeostasis. The depletion of Na^+^/H^+^ antiporter genes in HMS1 indicates that the *S*. *annulatum* endosymbiont is likely always sheltered in the host’s relatively stable internal environment. Furthermore, HMS1 lacks the CRISPR-cas system, consistent with the results of a previous study showing this system is missing in bacterial lineages adopting obligate endosymbiotic lifestyle.^58^ Together, all these genomic features indicate that the *S*. *annulatum* endosymbiont likely has evolved toward obligate endosymbiosis, extending our knowledge on the lifestyle and evolutionary process of siboglinid endosymbionts.

The highly completed HMS1 genome allows accurate enumeration of MGEs. Transposases are often the main components of TEs that catalyze the movement of transposons within and between genomes, and are particularly abundant in pathogens and newly host-restricted symbionts.^59^ Massive expansions of TEs have been reported to mediate the loss of unnecessary genes in the anglerfish symbiont genome.^51^ Phage elements could also promote prokaryotic genome evolution through gene disruption, duplication, transduction, or by acting as anchor points for major chromosomal rearrangements.^60^ The highly abundant MGEs, including prophages and TEs in HMS1 together with their high expression activity, indicate that the *S*. *annulatum* endosymbiont is undergoing MGEs-mediated reductive evolution. However, the genome size of HMS1 remains substantially larger than those of strictly vertically transmitted symbionts such as the vesicomyid endosymbiont (1.03 Mb)^53^ and the flatworm symbiont (1.34 Mb).^52^ Because highly abundant TEs and MGEs are characteristic of obligate symbionts in the early stage,^50^ we deduce that the *S*. *annulatum* endosymbiont probably remains in the early stage of genome erosion.

The *S*. *annulatum* endosymbiont is distinct from the vestimentiferan symbionts with regard to transmission mode, yet the ecological and evolutionary drivers of this difference remain unknown. The Haima cold seep has at least six disconnected seepage areas. The giant tubeworm *Paraescarpia echinospica* has been observed at three of these sites,^61^ whereas *S*. *annulatum* was only found at a site adjacent to the six active seeps.^29^ This suggests a relatively low requirement of reductive fluids by *S*. *annulatum* when compared with vestimentiferans. Multiple *S*. *annulatum* individuals inhabit together and form a clump covering a large seafloor area, which might allow for full utilization of reductive fluids from the underlying sediment for chemoautotrophic biosynthesis. This capacity to utilize a low flux of cold seep fluids for survival may indicate a low growth rate and long-life span of *S*. *annulatum*. Tubeworms, especially those inhabiting cold seeps, could live for more than 200 years,^62^ and symbionts are released only when the host tubeworm dies. The restricted release of symbionts could lead to a shortage of symbiont bacteria in ambient environments and reduce the survival probability of tubeworm holobionts.^62^ Therefore, there is a possibility that the potentially long life span of *S*. *annulatum* allows the hosts to evolve into more innate relationships with their endosymbionts.

Although bacteriophages are widely distributed in natural habitats, their roles in symbiosis remain largely unknown.^63^^,^^64^ Even more rarely reported are active phages infecting bacterial endosymbionts in the deep ocean.^65^^,^^66^ Our identification of proHMS1 within the *S*. *annulatum* endosymbiont undergoing a lytic cycle offers an opportunity to understand the tripartite interactions among the phage, the endosymbiont and the animal host. To defend against the invasion of foreign genetic elements, prokaryotes possess defense systems such as CRISPR-Cas, R-M and TA immune systems.^67^^,^^68^ The missing of CRISPR-Cas system and the high expression levels of the R-M and TA genes indicate that the *S*. *annulatum* endosymbiont mainly relies on innate immunity to defend against the invasion of foreign viral particles, including the lytic phage proHMS1 and other potential phages in the host’s trophosome environment.^19^ In addition, the highly expressed DNA methyltransferase HhaI gene of proHMS1 likely plays a counter-defense role in resisting defense systems of the symbiont like that happened in the deep-sea snail holobiont.^66^ Our work thus provides a new case of internal phage-bacterial interactions in deep-sea chemosymbiosis ecosystems.

The mechanism that activates the lytic cycle of the lysogenic proHMS1 remains unclear. It has been reported that the SOS response induced by DNA damage can activate prophages into a lytic cycle,^69^ and our work implies that the *lexA* and *recA* genes for the SOS response are highly expressed in the *S*. *annulatum* endosymbiont. As a main factor causing DNA damage and the SOS response,^34^ a high level of oxidative stress in the symbiont is implied by the highly expressed ROS scavenger genes (among the top 50 expressed genes) in HMS1. A transcriptomic study of *R*. *pachyptila* symbionts with different sizes revealed higher expression of ROS scavengers in symbionts with a small size, and suggested that the symbionts confront host-induced oxidative stress in their division stage.^70^ A recent study revealed that the complete genome of *Candidatus* Endoriftia persephone in the tubeworm *R*. *pachyptila* has two identical rRNA operons.^22^ HMS1 also has two rRNA operon copies that probably support its high growth rate and high frequency of cell division,^71^ which may cause a higher level of host-induced oxidative stress on the symbionts. Therefore, we propose a hypothesis that the tubeworm host-induced oxidative stress activates the lysogenic phage into a lytic cycle by triggering the SOS response. Because the relative abundance of endosymbionts is the highest in the proHMS1-missing *S*. *annulatum* individual, it is possible that the tubeworm host controls the density of the endosymbiont population and harvests nutrients from symbiotic chemoautotrophic bacteria through activating the lytic cycle of the prophages, resembling the role of WO phages infecting the symbiotic *Wolbachia* bacteria.^65^ However, further studies are required to test this hypothesis.

### Conclusions

In this study, we assembled *Ca*. Vondammii deminuti (HMS1) – the complete genome of the *S*. *annulatum* endosymbiont with characteristics of early genome erosion toward host-restricted lifestyle. Furthermore, we found a lytic phage of the *Sclerolinum* symbiont and proposed a hypothesis of the complex symbiont-phage-host interactions. Given that HMS1 is the only assembled symbiont genome in the *Sclerolinum* tubeworm genus, it remains uncertain whether genome reduction also occurs in the symbionts of other *Sclerolinum* species. Although the vestimentiferan symbionts have been experimentally proved to be horizontally transmitted, it remains uncertain whether the Frenulata sulfur-oxidizing bacteria are obligate or facultative symbionts. The heterotrophic symbionts of *Osedax* have been proposed to be acquired secondarily after the loss of the original sulfur-oxidizing symbiont, yet more efforts are needed to test this hypothesis.^25^ Thus, more representative symbionts of the *Sclerolinum, Osedax* and Frenulata lineages should be sequenced to provide a better understanding of the diversity of symbioses in Siboglinidae, and to illustrate the evolution of such symbioses that enabled them to thrive in various deep-sea chemosynthetic habitats. The reported deep-sea interactions of active phage with symbionts are not fully inllustrated, and thus require further analyses especially in detecting lytic prophage proHMS1 *in situ* and comparing the metatranscriptomic and proteomic data of tubeworm individuals with or without active prophages.

### Limitations of the study

A limitation of our sampling design was a lack of surrounding seawater and sediment samples of the *Sclerolinum* tubeworms, which prevented us from verifying the absence of the *Sclerolinum* endosymbiont in the environment. Because the slender *S*. *annulatum* resides inside a very fragile tube, we could not obtain eggs and larvae for detecting the presence of endosymbiont using the fluorescence *in situ* hybridization method. Thus, research in the future should aim to provide such direct evidence to verify the obligate endosymbiosis of *Ca*. Vondammii deminuti with the *S*. *annulatum* host. As our samples were fixed on the deck of the research vessel (R/V), the metatranscriptome data might have been affected by changes in temperature and water pressure during the sample collection process, and thus could not comprehensively reflect the *in-situ* expression of the ROS scavenger and LexA repressor genes. Meanwhile, the incomplete tubeworm bodies and the limited number of proHMS1-missing individuals could weaken the comparison of the relative abundance of endosymbionts among individuals. Besides, the evidence chain to prove the hypothesis of tubeworm host controlling symbiont population through the active phage remains incomplete. Therefore, further efforts are also needed to clarify the relationships among the tubeworm host, the prophage proHMS1 and the endosymbiont HMS1.

## STAR★Methods

### Key resources table

**Table:** 

REAGENT or RESOURCE	SOURCE	IDENTIFIER
**Chemicals, peptides, and recombinant proteins**		

RNAlater	Thermoisher Scientific	Cat#AM7021
Trizol	Invitrogen	Cat#15596026

**Deposited data**		

Raw metagenomic and metatranscriptomic data of the tubeworm *Sclerolinum annulatum*	This study	NCBI under BioProject PRJNA595466
The endosymbiont genome	This study	NCBI under accession number CP099567

**Software and algorithms**		

TRIMMOMATIC v0.38	Bolger et al., 2014^72^	N/A
BBmap	https://www.osti.gov/servlets/purl/1241166	N/A
Canu v1.8	Koren et al., 2017^73^	N/A
SPAdes v3.13	Bankevich et al., 2012^74^	N/A
MetaWrap v1.2.2	Uritskiy et al., 2018^75^	N/A
minimap2	Li, 2018^76^	N/A
Miniasm/Minipolish	Wick & Holt, 2019^77^	N/A
Pilon v.1.22	Walker et al., 2014^78^	N/A
Circlator 1.5.5	Hunt et al., 2015^79^	N/A
CheckM v1.1.0	Parks et al., 2015^80^	N/A
Prokka v1.11	Seemann, 2014^81^	N/A
BlastKOALA	Kanehisa et al., 2016^82^	N/A
eggNOG-mapper	Huerta-Cepas et al., 2019^83^	N/A
pfam_scan.pl	Punta et al., 2012^84^	N/A
PHAST web server	Zhou et al., 2011^85^	N/A
CoverM 0.4.0	https://github.com/wwood/CoverM	N/A
Meta_RNA	Huang et al., 2009^86^	N/A
MAFFT L-INS-i v7.294b	Katoh & Toh, 2010^87^	N/A
Bandage 0.8.1	Wick et al. 2015^88^	N/A
trimAl v1.4	Capellagutiérrez et al., 2009^89^	N/A
raxmlGUI v1.5	Silvestro & Michalak, 2012^90^	N/A
pyani	https://pypi.org/project/pyani/	N/A

### Resource availability

#### Lead contact

Further information and requests for resources and reagents should be directed to and will be fulfilled by the lead contact, Yong Wang (wangyong@sz.tsinghua.edu.cn).

#### Materials availability

All unique/stable reagents in this study are available from the lead contact without restriction.

### Experimental model and subject details

No experimental models were used in this study.

### Method details

#### Sample collection

Specimens of *S*. *annulatum* were collected from the Haima cold seep (depth 1,425 m) in the South China Sea^29^ using ROV *Haima* on board the R/V *Haiyang* 6 in May 2019. Because the slender *S*. *annulatum* (i.e., diameter <0.6 mm) resides inside a very fragile tube, all of the sampled individuals were incomplete. Upon arrival at the main deck of the R/V, specimens were rinsed several times with 0.22 μm membrane-filtered iced seawater to remove sediment, debris, and microbes attached to their tubes. The cleaned specimens were either frozen immediately at −80°C or preserved in 100% ethanol for metagenomic analyses, or fixed in RNAlater (Thermoisher Scientific, Waltham, MA, USA) and later stored at −80°C for metatranscriptomic analyses. All the specimens were embedded in dry ice and transported to the laboratory at the end of the cruise.

#### DNA extraction

The slenderness of *S*. *annulatum* made it difficult to dissect enough intact soft tissues for DNA extraction. Therefore, DNA was extracted from tubeworm individuals along with their tubes. Individual HM_W22 (tube length 50.2 cm) was subjected to DNA extraction using the CTAB method^91^ for both Illumina and PacBio sequencing (Figure S10). A batch of mixed individuals was rinsed with 100% ethanol and then dissected into the pale white anterior part (HM_W02) and the brownish posterior part (HM_W03) for creating datasets with different sequencing coverages and facilitating genome binning (Figures S10 and S11). Both samples were further fragmented with sterile scissors and subjected to DNA extraction using the PowerSoil DNA isolation kit (MoBio, Carlsbad, USA) according to the manufacturer’s protocol. Genomic DNA was also extracted from ten other individuals (HM_W11–HM_W20) using the PowerSoil DNA isolation kit for relative abundance analysis (Figure S10). DNA purity was measured using a NanoDrop ND-1000 spectrophotometer (Thermo Fisher Scientific, Wilmington, USA). DNA quality and quantity were checked using gel electrophoresis and a Qubit 2.0 Fluorometer (Invitrogen, USA).

#### Metagenome sequencing

Metagenomic libraries of samples HM_W02 and HM_W03 with an insert size of 550 bp were separately constructed following the standard protocol of the Illumina TruSeq Nano DNA Sample Prep Kit (Illumina, USA), and sequenced on an Illumina HiSeq sequencer at Berry Genomics Co. Ltd. (Beijing, China) to produce 150 bp paired-end reads. Metagenomic libraries of the individuals HM_W11 to HM_W20 and HM_W22 with an insert size of 350 bp were constructed following the standard protocol of the NEBNext DNA Library Prep Kit (NEB, USA) and sequenced on an Illumina NovaSeq 6000 sequencer at Novogene Co. Ltd. (Beijing, China) to produce 150 bp paired-end reads. Illumina reads were trimmed using TRIMMOMATIC v0.38^72^ with the following parameters: LEADING = 10, TRAILING = 10, SLIDINGWINDOW = 4:15, and MINLEN = 40. Reads of HM_W22 were further normalized using the bbnorm.sh script of BBmap^92^ with a depth normalization setting of 30X (target = 30). A 20 kb SMRTbell library of individual HM_W22 (named HM_W22P) was also constructed and sequenced on a PacBio Sequel platform at Novogene Co. Ltd. (Beijing, China). PacBio reads were corrected and trimmed using Canu v1.8^73^ to retain reads ≥15,000 bp. Overall, a total of 13 Illumina, along with one PacBio dataset, were obtained for downstream metagenomics analyses (Table S4).

#### Metagenome assembly and genome binning

The metagenome assembly strategy is illustrated in Figure S10. Firstly, the filtered and cleaned Illumina and PacBio reads of HM_W22 were co-assembled using SPAdes v3.13^74^ with a *k*-mer set of 21, 33, 55, 77, 99 and 127 under the “--only-assembler” mode. The assembled metagenome was subjected to genome binning using MetaWrap v1.2.2 under the default settings,^75^ with the Illumina datasets HM_W02, HM_W03 and HM_W22 being used in the “Binning” module to provide distinct coverages for the metagenomic contigs and to improve the binning quality. A high-quality draft genome (binC) was extracted from the co-assembled metagenome. Secondly, PacBio reads were remapped to binC using minimap2,^76^ and the mapped reads were reassembled using the “Miniasm/Minipolish” pipeline^77^ with results visualized using Bandage 0.8.1.^88^ The genome assembly consisted of two long contigs and two variable regions. One of the variable regions is an unclosed gap caused by a fragment with unusually high coverage (region R1), and the other is an unsolved cross-interconnected region (region R2) (Figure S12A). The Illumina reads mapped to R1 were extracted from HM_W22 and reassembled by SPAdes v3.13, which produced a circular phage genome for proHMS1 (Figure 1D). The reads coverage on the phage region is much higher than the coverage on the endosymbiont genome (Figure S8). Alignment of long PacBio reads reveals that proHMS1 also could be integrated into the endosymbiont genome, suggesting that proHMS1 has both the lysogenic and lytic forms, representing an active prophage (Figure S8). When we tried to assemble raw PacBio reads of HM_W22 by the “Miniasm/Minipolish” pipeline, a linear contig could solve the cross interconnection of region R2 (Figure S12B). Therefore, we manually merged the two long contigs (Figure S12A) with the prophage region R1 and the linear region R2 to produce a finished genome for the endosymbiont (HMS1, Figure 1C). The genome was further polished using Pilon v.1.22^78^ with three iterations using the paired-end Illumina short clean reads. The genome start position was determined using the “fixstart” module in Circlator 1.5.5.^79^

Besides, the endosymbiont genome of *S*. *annulatum* was assembled using SPAdes v3.13 based on Illumina short clean reads of HM_W02 and HM_W03. The assembled metagenome was subjected to genome binning using MetaWrap v1.2.2 under the default settings, which produced the draft genome binI. Assembly graphs produced by SPAdes were explored with Bandage 0.8.1 and 150 nodes with length of longer than 2,000 bp were entirely consistent with the contigs of genome binI (Figure S13). To determine variations among individuals, metagenomic assembly and genome binning were separately carried out using the Illumina short clean reads for HM_W11 to HM_W20. Genome completeness and contamination were evaluated using CheckM v1.1.0.^80^

#### Genome annotation

All the assembled genomes were automatically annotated using Prokka v1.11 under the default settings.^81^ Signal peptides were identified from the Prokka annotation. The predicted amino acid sequences were annotated by using the Kyoto Encyclopedia of Genes and Genomes (KEGG), Clusters of Orthologous Groups (COGs) and PFAM databases. KEGG annotation was performed using the online BlastKOALA program against the species-level prokaryotic KEGG gene database.^82^ COG annotation was performed using the eggNOG-mapper tool against the eggNOG v5.0 database.^83^ PFAM annotation was performed using the script pfam_scan.pl by searching against the PFAM 32.0 database.^84^ Functional annotations of the three bins (binC, HMS1 and binI) based on KEGG/COG/PFAM annotations produced highly consistent results, indicating the high quality of the finished endosymbiont genome of *S*. *annulatum* and the reliability of our annotation results (Figure S14 and Table S21. Functional comparisons of three genomic bins of the Sclerolinum annulatum endosymbionts based on annotation against the KEGG database, related to STAR Methods, Table S22. Functional comparisons of three genomic bins of the Sclerolinum annulatum endosymbionts based on annotation against the COG database, related to STAR Methods, Table S23. Functional comparisons of three genomic bins of the Sclerolinum annulatum endosymbionts based on annotation against the PFAM database, related to STAR Methods). The vestimentiferan endosymbionts and *Sedimenticola* species used for comparative analyses were re-annotated using the above methods. Principal components analysis (PCA) was performed to cluster the endosymbiont and the free-living genomes based on their KEGG/COG/PFAM annotations according to a method used in our recent study.^93^ Potential prophages were predicated using the PHAST web server.^85^

#### Metatranscriptome sequencing and analyses

Three individuals of *S*. *annulatum* (HM_W26, HM_W27 and HM_W28) were used for total RNA extraction using the Trizol reagent (Invitrogen, USA) following the manufacturer’s protocol. Quality and quantity of the extracted RNA were examined using 1% gel electrophoresis, and a Qubit 2.0 Fluorometer (Invitrogen, Carlsbad, USA), respectively. Qualified total RNA was then sent to Novogene Co. Ltd. (Beijing, China) for metatranscriptomic sequencing. Briefly, ribosomal RNA was removed from the total RNA of each individual using the NEBNext Ultra RNA Library Prep Kit for Illumina (NEB, Ipswich, USA). The resultant mRNA was reverse-transcribed into cDNA and then fragmented into 250 to 300 bp. The constructed libraries were afterward sequenced on an Illumina NovaSeq 6000 sequencer (Illumina, San Diego, USA) to produce 150 bp paired-end reads. Adapters and low-quality reads were removed using Trimmomatic v.0.38^72^ under the following settings: LEADING = 10, TRAILING = 10, SLIDINGWINDOW = 4:15, MINLEN = 40. Clean reads for each individual were then mapped to the endosymbiont genome with BBmap^92^ with a minimum identity of 0.98. Reads counts for symbiont genes in each individual were calculated with CoverM 0.4.0 (https://github.com/wwood/CoverM) and normalized with the transcripts per million calculation (TPM) values.^94^ Average TPM values (TPM_av_) across the three tubeworm individuals were summarized for the estimated gene expression levels.

#### Phylogenetic analyses

The 16S rRNA gene sequence of the *S*. *annulatum* endosymbiont was identified using Meta_RNA under the default settings.^86^ For phylogenetic analysis, the 16S rRNA gene sequence was queried against the NCBI GenBank database using BLASTN to recruit sequences of close relatives. These sequences were aligned using MAFFT L-INS-i v7.294b,^87^ and the poorly aligned regions were trimmed using trimAl v1.4 under the -automated1 setting.^89^ A maximum-likelihood (ML) tree was constructed using raxmlGUI v1.5^90^ with the GTRGAMMA model for 1,000 replicates. For phylogenomic analyses, available tubeworm endosymbiont genomes were downloaded from the NCBI genome database. In addition, the 16S rRNA gene sequence of the *S*. *annulatum* endosymbiont was searched against the NCBI genome database to find the other close relative genomes. Concatenated alignment sequences of 43 conserved proteins were derived from these genomes using CheckM v1.1.0 program^80^ and further treated using trimAl v1.4 under the default settings. An ML tree was built using raxmlGUI v1.5 with the PROTGAMMALG model for 100 replicates. The resulting trees were visualized using the Interactive Tree Of Life (iTOL) v5.^95^ Average nucleotide identity (ANI) between genomes was calculated using pyani (https://pypi.org/project/pyani/).

### Quantification and statistical analysis

Three metatranscriptomic datasets from separated individuals were used to evaluate the consistence of gene expression among biological replicated using the Pearson correlation coefficient (Results and Figure S4). nMDS ordination of HMS1 and reference genomes was performed based on the relative abundance of their KOs using the Bray-Curtis distance in PRIMER-E. The two-dimensional stress value of 0.049 indicate good fit (Figure 3A). Other statistical details are provided in the corresponding figure legends.

## Data Availability

Raw metagenomic and metatranscriptomic reads are deposited in the NCBI Sequence Reads Archive (SRA) database under BioProject PRJNA595466. The endosymbiont genome is deposited in the NCBI GenBank database under the genome accession number CP099567. This paper does not report original code. Additional information required to reanalyze the data in this paper is available from the lead contact upon request.

## Supplemental information

Document S1. Note S1 and Figures S1–S14

Table S1. Detail genomic information of the Sclerolinum annulatum endosymbiont HMS1 and its relatives, related to Figure 1C and Table 1

Table S2. Reference genomes in Figure 2A

Table S3. Average Nucleotide Identity (ANI) calculations for the Sclerolinum annulatum endosymbiont HMS1 and its relatives, related to Figure 2B

Table S4. High-throughput sequencing information of metagenomes and metatranscriptomes, related to STAR Methods

Table S5. Transcriptomic expression of endosymbiont genes in three Sclerolinum annulatum individuals, related to Figures 4B and S4

Table S6. Genomic quality evaluation of the Sclerolinum annulatum endosymbiont HMS1 and its relatives by the CheckM program, related to Table 1

Table S7. Genes involved in selected metabolism pathways of the Sclerolinum annulatum endosymbiont HMS1 and its relatives, related to Figure 4A

Table S8. Genes encoding transporters in the Sclerolinum annulatum endosymbiont HMS1 and its relatives, related to Figure 4A

Table S9. Genes encoding two-component systems in the Sclerolinum annulatum endosymbiont HMS1 and its relatives, related to Figure 4A

Table S10. Genes encoding motility machines and chemotaxis proteins in the Sclerolinum annulatum endosymbiont HMS1 and its relatives, related to Figure 4A

Table S11. Genes encoding secretion systems in the Sclerolinum annulatum endosymbiont HMS1 and its relatives, related to Figure 4A

Table S12. Genes encoding phage-coding proteins in the Sclerolinum annulatum endosymbiont HMS1 and its relatives, related to Figure 1D

Table S13. Summary of prophages in the Sclerolinum annulatum endosymbiont HMS1 genome predicted by the Phaster online program, related to Figure 1C

Table S14. Transcriptomic expression of phage-coding proteins in the Sclerolinum annulatum endosymbiont HMS1, related to Figure 4B

Table S15. Genes encoding transposases in the Sclerolinum annulatum endosymbiont HMS1 and its relatives, related to Figure 1C

Table S16. Transposable elements in the Sclerolinum annulatum endosymbiont HMS1 predicted by the ISfinder, related to Figure 1C

Table S17. Transposase genes of the Sclerolinum annulatum endosymbiont HMS1 and their expression, related to Figures 1C and 4B

Table S18. Functional annotation and transcriptomic expression of the prophage proHMS1, related to Figure 1D and STAR Methods

Table S19. Relative abundance of the Sclerolinum annulatum endosymbiont HMS1 and prophage proHMS1 among different individuals, related to STAR Methods

Table S20. Genes encoding prokaryotic defense systems in the Sclerolinum annulatum endosymbiont HMS1 and its relatives, related to Figure 4A

Table S21. Functional comparisons of three genomic bins of the Sclerolinum annulatum endosymbionts based on annotation against the KEGG database, related to STAR Methods

Table S22. Functional comparisons of three genomic bins of the Sclerolinum annulatum endosymbionts based on annotation against the COG database, related to STAR Methods

Table S23. Functional comparisons of three genomic bins of the Sclerolinum annulatum endosymbionts based on annotation against the PFAM database, related to STAR Methods
