# Supplementary material for: Early genome erosion and internal phage-symbiont-host interaction in the endosymbionts of a cold-seep tubeworm
Source: iScience. 2023 Jun 7;26(7):107033. doi: 10.1016/j.isci.2023.107033 (PMC10300362; doi:10.1016/j.isci.2023.107033)
Supplement: Document S1. Note S1 and Figures S1–S14 [file mmc1.pdf]

iScience, Volume *26*

Supplemental information

Early genome erosion and internal phage-symbiont-host interaction in the endosymbionts of a cold-seep tubeworm

Zhao-Ming Gao, Ting Xu, Hua-Guan Chen, Rui Lu, Jun Tao, Hong-Bin Wang, Jian-Wen Qiu, and Yong Wang

Note S1. Secretion systems, related to Figure 4A.

Genes of tubeworm endosymbionts encoding secretion machines and their transcriptomic expression are summarized in Additional file 2: Table S11. All the vestimentiferan endosymbionts contain a type II secretion system (T2SS) that is supposed to export hemolysins and chitinases for the symbionts to permeabilize into host cells [1], but HMS1 lost it. However, HMS1 has a *lapEBC* gene cluster encoding the T1SS adhesion protein transporter system, which has also been found in the MCR genomes. It is likely that *Sclerolinum* endosymbiont secrets hemolysin as its adhesion protein through the alternative T1SS secretion pathway, which helps the newly generated symbionts to enter host cells. The expression levels of *lapEBC* transporter genes are relatively low with TPM_{av} values ranging from 21.48 to 52.07, indicating their very limited roles for the endosymbiont.

The T4SS system is present in many groups of bacteria and can secrete a wide range of substrates from single proteins to protein-protein and protein-DNA complexes [2]. As one of their functions, T4SS can deliver effector macromolecules into eukaryotic cells during the infection progress of pathogenic bacteria and mediate the injection of virulence proteins into mammalian host cells [2]. T4SS is needed for animal pathogens to establish and maintain themselves in the proper vacuolar compartment [3,4]. HMS1 also has genes encoding the type IV conjugal DNA-protein transfer (VirB/D) proteins (T4SS), which are not found in the vestimentiferan endosymbionts. However, the T4SS in HMS1 is quite incomplete compared to the functional *vir* systems [5]. In addition, the expression levels of T4SS genes in HMS1 are quite low with TPM_{av} values ranging from 7.91 to 15.74, and therefore, the function of T4SS in HMS1 is considered to be limited.

Type VI Imp/Vas secretion system (T6SS) is the most recently described bacterial secretion system that assembles into a bacteriophage tail-like structure and can directly transport effector proteins into target prokaryotic or eukaryotic cells for pathogenesis or competitive survival [6,7]. Thirteen core genes of T6SS Imp/Vas secretion system have been found in the vestimentiferan endosymbionts of *Escarpia laminata*, *Lamellibrachia barhami*, *Galathealinum brachiosum*, and *Paraescarpia echinospica*. However, only seven core genes were found in the HMS1 genome, indicating an incomplete T6SS Imp/Vas secretion system. Due to the relatively low expression levels of the T6SS genes (ranging from 17.04 to 235.64), they may not play a vital role in HMS1.

References

1. Li, Y.N., Liles, M.R., and Halanych, K.M. (2018). Endosymbiont genomes yield clues of tubeworm success. ISME J. *12*, 2785-2795. https://doi.org/10.1038/s41396-018-0220-z.

2. Fronzes, R., Christie, P.J., and Waksman, G. (2009). The structural biology of type IV secretion systems. Nat Rev Microbiol *7*, 703-714. https://doi.org/10.1038/nrmicro2218.

3. Jones, K.M., Lloret, J., Daniele, J.R., and Walker, G.C. (2007). The type IV secretion system of *Sinorhizobium meliloti* strain 1021 is required for conjugation but not for intracellular symbiosis. J Bacteriol *189*, 2133-2138. https://doi.org/10.1128/JB.00116-06.

4. Schulein, R., and Dehio, C. (2002). The VirB/VirD4 type IV secretion system of *Bartonella* is essential for establishing intraerythrocytic infection. Mol Microbiol *46*, 1053-1067. https://doi.org/10.1046/j.1365-2958.2002.03208.x.

5. Sgro, G.G., Oka, G.U., Souza, D.P., Cenens, W., Bayer-Santos, E., Matsuyama, B.Y., Bueno, N.F., Dos Santos, T.R., Alvarez-Martinez, C.E., Salinas, R.K., and Farah, C.S. (2019). Bacteria-Killing Type IV Secretion Systems. Front Microbiol *10*, 1078. https://doi.org/10.3389/fmicb.2019.01078.

6. Coulthurst, S.J. (2013). The Type VI secretion system - a widespread and versatile cell targeting system. Res Microbiol *164*, 640-654. https://doi.org/10.1016/j.resmic.2013.03.017.

7. Lien, Y.W., and Lai, E.M. (2017). Type VI secretion effectors: methodologies and biology. Front Cell Infect Mi *7*, 254. https://doi.org/ARTN 254
10.3389/fcimb.2017.00254.

Supplementary Figures

```
16S_rRNA-1   100
16S_rRNA-2   100
Consensus    gtttgatcctggctcagattgaacgctggcggtatgcttaacacatgcaagtcgaacggtaacagaggagagcttgctctcctgctgacgagtggcggac

16S_rRNA-1   200
16S_rRNA-2   200
Consensus    gggtgagtaacgcgtaggaatctgcctagtagtgggggacaacccgaggaaactcgggctaataccgcatacgccctatgggggaaagcaggggatcttc

16S_rRNA-1   300
16S_rRNA-2   300
Consensus    ggaccttgcgctattagatgagcctgcgtcggattagctagttggtgaggtaatggctcaccaaggcgacgatccgtagctggtctgagaggacgatcag

16S_rRNA-1   400
16S_rRNA-2   400
Consensus    ccacactgggactgagacacggcccagactcctacgggaggcagcagtggggaatattggacaatgggggcaaccctgatccagcaataccgcgtgtgtg

16S_rRNA-1                        G                                             A                                  500
16S_rRNA-2                        A                                             C                                  500
Consensus    aagaaggcctgcgggttgtaaagcactttcaattgtgaagaaaagcttgggattaatactctcgggtattgacgttaactttagaagaagcaccggctaa

16S_rRNA-1   600
16S_rRNA-2   600
Consensus    ccctgtgccagcagccgcggtaatacaggggggtgcgagcgttaatcggaattactgggcgtaaagcgtgcgtaggcggcttgttcagtcaggtgtgaaag

16S_rRNA-1   700
16S_rRNA-2   700
Consensus    ccccgggctcaacctgggaattgcatttgatactagcaggctagagtatggtagagggaagtggaattccgggtgtagcggtgaaatgcgtagatatccg

16S_rRNA-1   800
16S_rRNA-2   800
Consensus    gaggaacaccagtggcgaaggcggcttcctggaccaatactgacgctgaggtacgaaagcgtgggtagcaaacaggattagataccctggtagtccacgc

16S_rRNA-1   900
16S_rRNA-2   900
Consensus    tgtaaacgatgtcaactagccgttgggggctttaattgtctttagtggcgcagctaacgcaataagttgaccgcctggggagtacggccgcaaggttaaa

16S_rRNA-1   1000
16S_rRNA-2   1000
Consensus    actcaaaggaattgacgggggcccgcacaagcggtggagcatgtggtttaattcgatgcaacgcgaagaaccttaccagctcttgacatcctgcgaactt

16S_rRNA-1   1100
16S_rRNA-2   1100
Consensus    tctagagatagaaaggtgccttcgggaacgcagtgacaggtgctgcatggctgtcgtcagctcgtgtcgtgagatgttgggttaagtcccgtaacgagcg

16S_rRNA-1   1200
16S_rRNA-2   1200
Consensus    caacccttgtccttagttgccagcgcgtaatggcgggaactctagggagactgccggtgataaaccggaggaaggtggggatgacgtcaagtcatcatgg

16S_rRNA-1   1300
16S_rRNA-2   1300
Consensus    cccttatgagctgggctacacacgtgctacaatggccggtacagagggctgcaacctcgtgagggtgagcgaatctcataaagccggtcgtagtccggat

16S_rRNA-1   1400
16S_rRNA-2   1400
Consensus    tggagtctgcaactcgactccatgaagtcggaatcgctagtaatcgcgaatcagaatgtcgcggtgaatacgttcccgggccttgtacacaccgcccgtc

16S_rRNA-1   1500
16S_rRNA-2   1500
Consensus    acaccatgggagtgggttgcaaaagaagtgggtagtctaaccttcgggaggacgctcaccactttgtgattcatgactggggtgaagtcgtaacaaggta

16S_rRNA-1   1544
16S_rRNA-2   1544
Consensus    gccctaggggaacctggggctggatcacctcctttaaagaaagc
```

Figure S1. Alignment of two 16S rRNA gene copies of the *Sclerolinum annulatum* endosymbiont HMS1, related to Figure 1C.

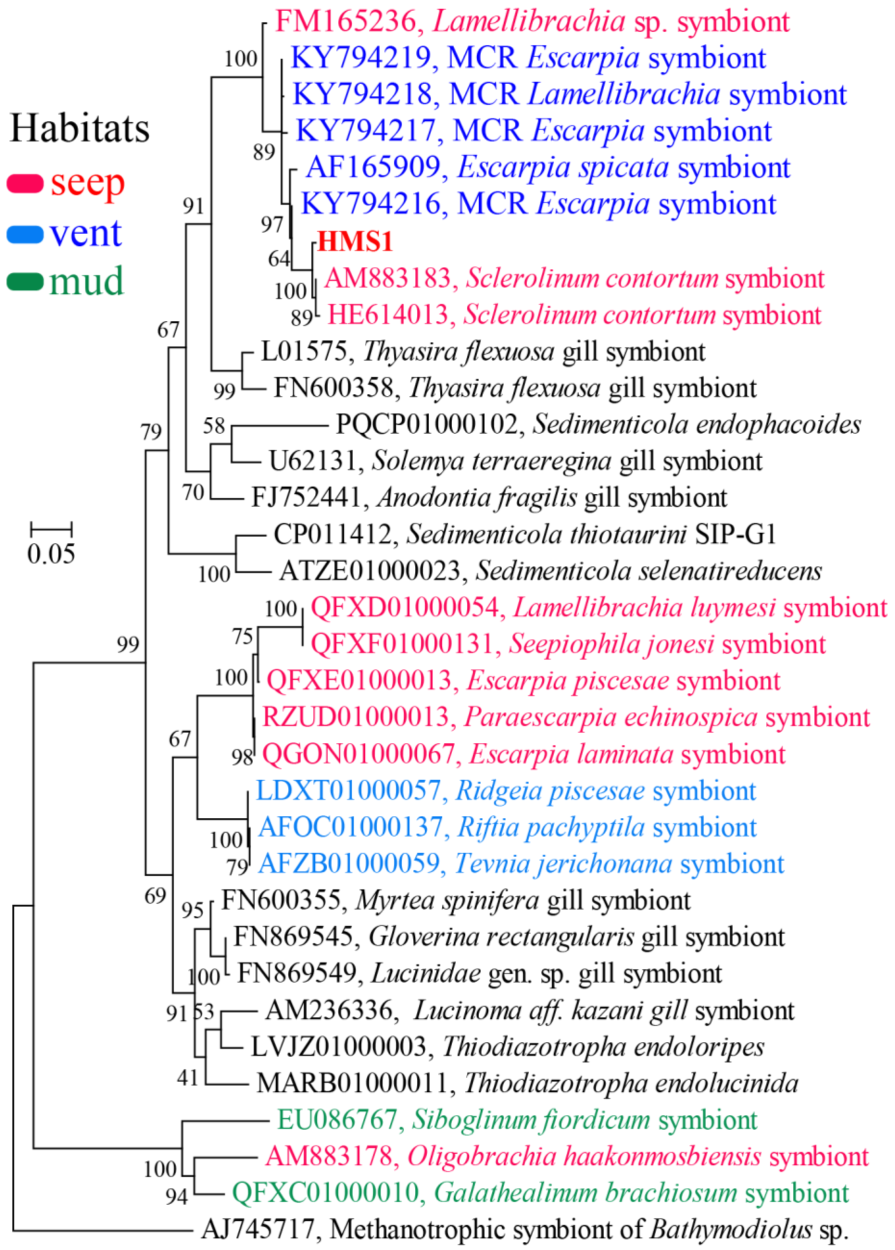

Figure S2. Phylogenetic tree of the *Sclerolinum annulatum* endosymbiont HMS1, related to Figure 2A. Maximum-likelihood tree based on partial 16S rRNA gene sequences (1,495 bp) was constructed with the GTRGAMMA model for 1,000 bootstraps

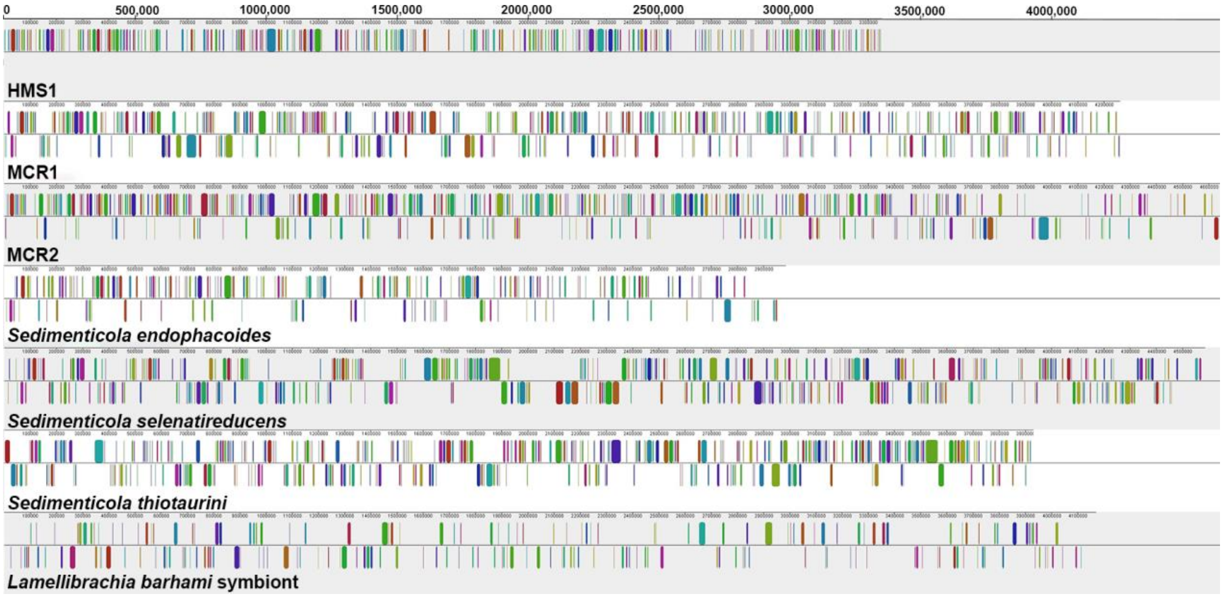

Figure S3. Genome alignment of the *Sclerolinum annulatum* endosymbiont HMS1 and its close relatives, related to Figure 2A. Colored collinear blocks indicate homologous sequences with no structural variations. Inverted fragments are shown under the horizontal line. HMS1 was used as the reference. The analyses were performed by the Mauve program**.**

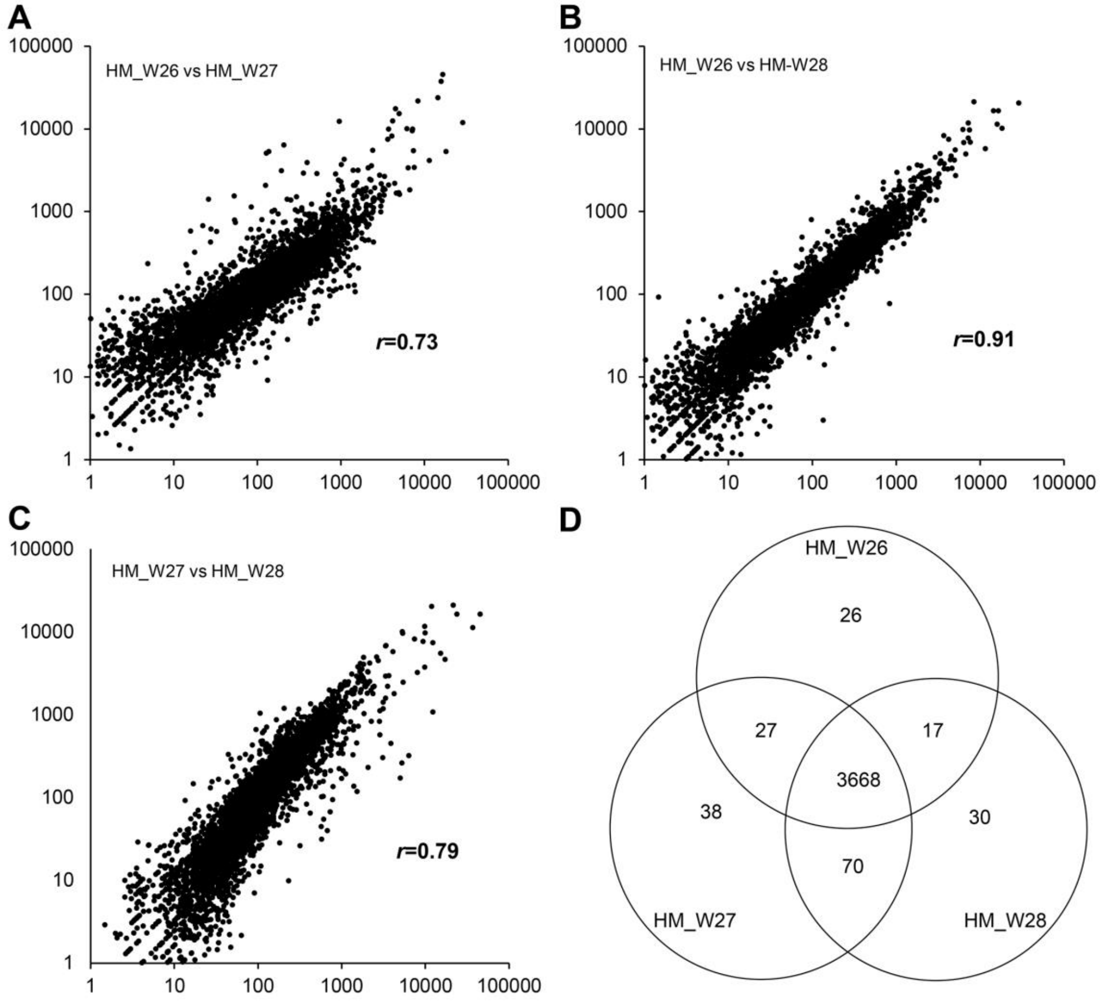

Figure S4. Gene expression of the *Sclerolinum annulatum* endosymbiont HMS1 in three individuals, related to Figure 4B. A-C Plots represent the correlation of transcript per million (TPM) values calculated from reads of three biological metatranscriptomes mapping to the genome HMS1. **D** Venn diagram showing the expressed genes of HMS1 in three *S. annulatum* individuals. *r* represents the Pearson correlation coefficient. Axes were transformed to a log base 10 scale.

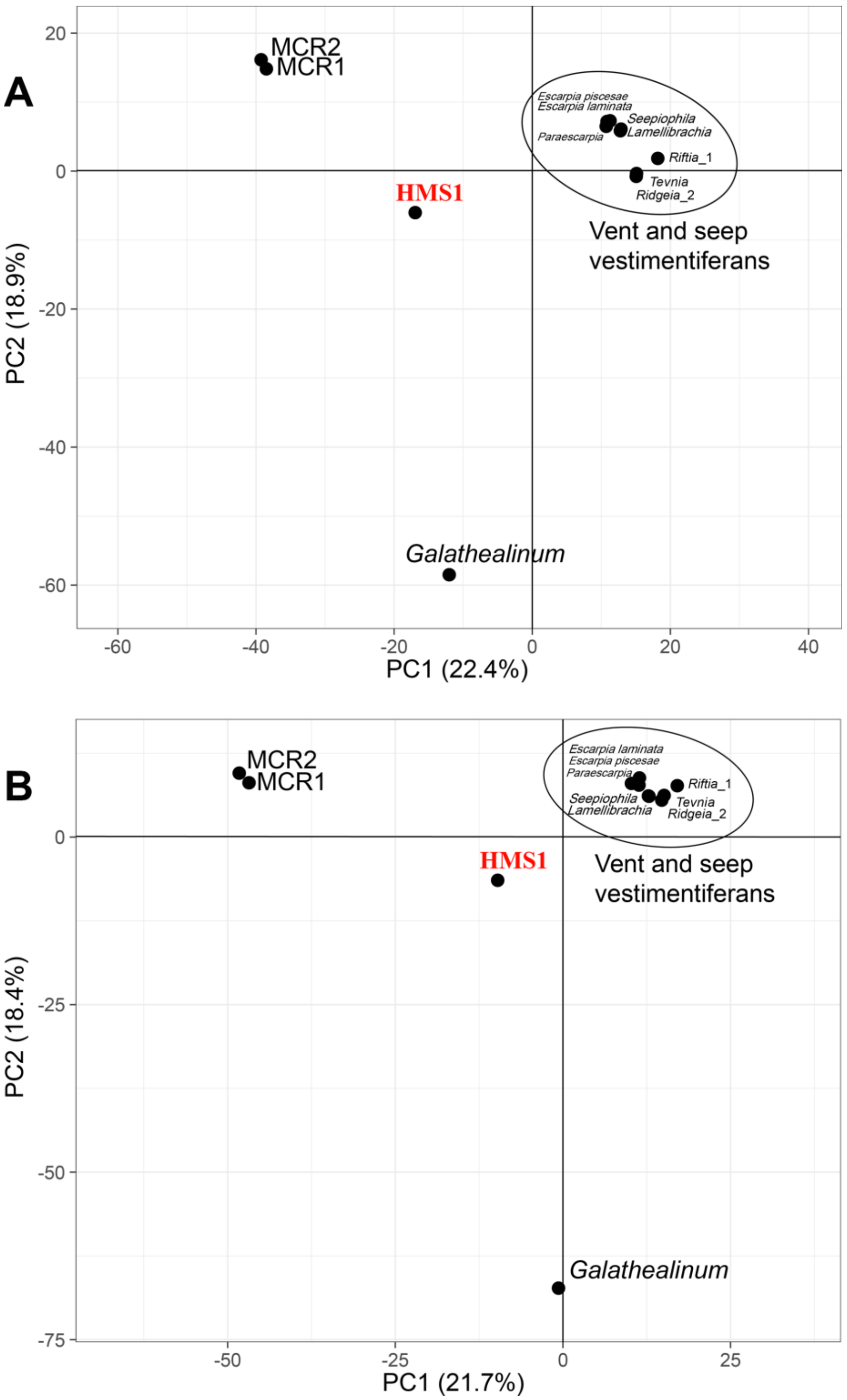

Figure S5. Functional clustering of the siboglinid tubeworm endosymbionts, related to Figure 3A. PCA plots were performed based on the COG (**A**) and PFAM (**B**) annotations.

Symbiont	HMS1	MCR1	MCR2	*Paraescarpia echinospica*	*Seepiophila jonesi*	*Lamellibrachia luymesi*	*Lamellibrachia barhami*	*Escarpia laminata*	*Escarpia piscesae*	*Riftia pachyptila*	*Ridgeia piscesae*	*Tevnia jerichonana*	*Galathealinum brachiosum*	*Sedimenticola thiotaurini*	*Sedimenticola selenatireducens*	*Sedimenticola endophacoides*	
H_2	0	2	1	1	1	1	1	1	1	1	1	1	1	3	3	2	*hyaB*
	0	2	1	1	1	1	1	1	1	1	1	1	2	3	5	2	*hyaA*
V-ATPase	0	1	1	2	2	2	2	2	2	4	2	2	0	1	1	1	*atpA*
	0	1	1	2	2	2	2	2	2	2	2	2	0	1	1	1	*atpB*
	0	1	1	1	1	1	1	1	1	1	1	1	0	1	1	1	*atpC*
	0	1	1	2	2	2	2	2	2	2	2	2	0	1	1	1	*atpD*
	0	1	1	2	2	2	2	2	2	2	2	2	0	1	1	1	*atpE*
	0	1	1	3	3	3	3	3	3	3	2	2	0	2	2	2	*atpI*
	0	1	1	2	2	2	2	2	2	2	2	2	0	1	1	1	*atpK*
Nitrogen	0	0	0	0	1	1	0	0	0	2	1	1	0	1	2	0	*narG*
	0	0	0	0	1	1	0	0	0	1	1	1	0	2	3	0	*narH*
	1	0	0	1	2	2	1	1	1	2	2	2	1	2	2	1	*narI*
	0	2	1	1	1	1	1	2	2	1	1	1	1	2	1	2	*nirS*
	0	1	1	1	1	1	1	1	1	0	1	1	1	2	2	1	*norB*
	0	1	1	1	2	2	1	1	1	0	1	1	1	2	2	1	*norC*
	0	1	1	1	1	1	2	1	1	1	1	1	1	1	1	1	*nosZ*
Vitamin B12	0	0	1	1	1	1	1	1	1	1	1	1	0	1	1	0	*cobA*
	0	1	1	0	0	0	0	0	0	1	1	1	1	1	1	0	*cobQ*
	0	1	1	0	1	1	0	0	0	0	0	0	0	1	0	0	*cbiB*
	0	1	1	0	0	0	0	0	0	0	0	0	0	1	0	0	*cobC1*
	0	1	1	0	1	1	0	1	1	1	1	1	1	1	1	0	*cobP*
	0	1	1	0	1	1	0	1	0	1	1	1	1	1	1	0	*cobS*
	0	1	1	0	2	2	0	1	1	1	1	1	1	2	1	0	*cobU*
	0	1	1	1	2	2	1	2	1	2	2	2	2	1	1	1	*cobC*
Vitamin K2	0	1	1	1	1	1	1	1	1	1	1	1	0	1	1	1	*menF*
	0	1	1	1	1	1	1	1	1	1	1	1	0	1	1	2	*menD*
	0	1	1	1	1	1	1	1	1	1	1	1	0	1	1	1	*menE*
	0	1	1	1	1	1	1	1	1	1	1	1	0	1	1	1	*menB*
Na+/H+ antiporters	0	1	1	2	1	1	2	1	1	1	1	1	1	0	0	2	*mnhB*
	0	1	1	2	1	1	2	1	1	0	1	1	1	0	0	2	*mnhC*
	0	0	0	3	1	1	3	1	1	1	1	1	3	0	0	3	*mnhD*
	0	1	1	3	1	1	3	1	1	1	1	1	1	0	0	2	*mnhE*
	0	1	1	2	1	1	2	1	1	1	1	1	1	0	0	2	*mnhF*
	0	1	1	2	1	1	2	1	1	1	1	1	1	0	0	2	*mnhG*
TCSs	0	2	3	3	1	1	4	3	2	2	3	3	1	1	6	0	*envZ*
	0	1	2	2	1	1	2	2	2	2	3	2	1	0	3	0	*ompR*
	0	1	1	1	0	0	1	1	1	1	1	1	0	1	1	1	*qseC*
	0	1	1	1	0	0	1	1	0	1	1	1	0	1	1	2	*qseB*
	0	1	1	1	1	1	1	1	1	1	1	1	1	1	1	1	*flrB*
	0	1	1	1	1	1	1	1	1	1	1	1	1	1	1	1	*flrC*
	0	2	2	2	2	2	2	2	2	2	3	3	0	2	2	0	*rpfC*
	0	1	1	1	1	1	1	1	1	1	1	1	0	1	2	0	*rpfG*
Motility	2	33	58	37	36	36	39	40	43	35	37	35	39	20	28	16	**Chemotax**
	2	51	52	46	36	36	46	48	48	48	49	50	48	51	55	54	**Flagellar**
	23	21	21	26	19	19	17	17	20	23	20	21	18	19	17	19	**Pilus**
	8	10	10	10	12	11	11	10	10	12	11	10	13	10	11	9	**Twitching**
	0	1	1	1	1	1	2	1	1	1	1	1	1	1	1	1	*pilR*
	0	1	1	1	1	1	1	1	1	1	1	1	1	1	1	1	*pilS*
Secretion systems	1	29	17	30	14	14	28	28	29	28	27	27	19	3	15	2	**T2SS gene**
	2	1	2	0	0	0	0	0	0	0	0	0	0	0	0	6	*lapB*
	4	1	1	0	0	0	0	0	0	0	0	0	0	0	0	6	*lapC*
	2	1	1	0	0	0	0	0	0	0	0	0	0	0	0	2	*lapE*
	2	0	0	0	0	0	0	0	0	0	0	0	0	0	0	0	*virB5*
	1	0	0	0	0	0	0	0	0	0	0	0	0	0	0	0	*virB6*
	1	0	1	0	1	1	0	0	0	0	0	0	0	0	0	0	*virD4*
	11	0	2	20	3	3	19	20	10	2	12	3	18	1	2	0	**T6SS**

Figure S6. Functional divergence of the *Sclerolinum annulatum* endosymbiont HMS1 compared to its relatives, related to Figure 4A. Comparative analysis was based on functional annotations against the KEGG/COG databases.

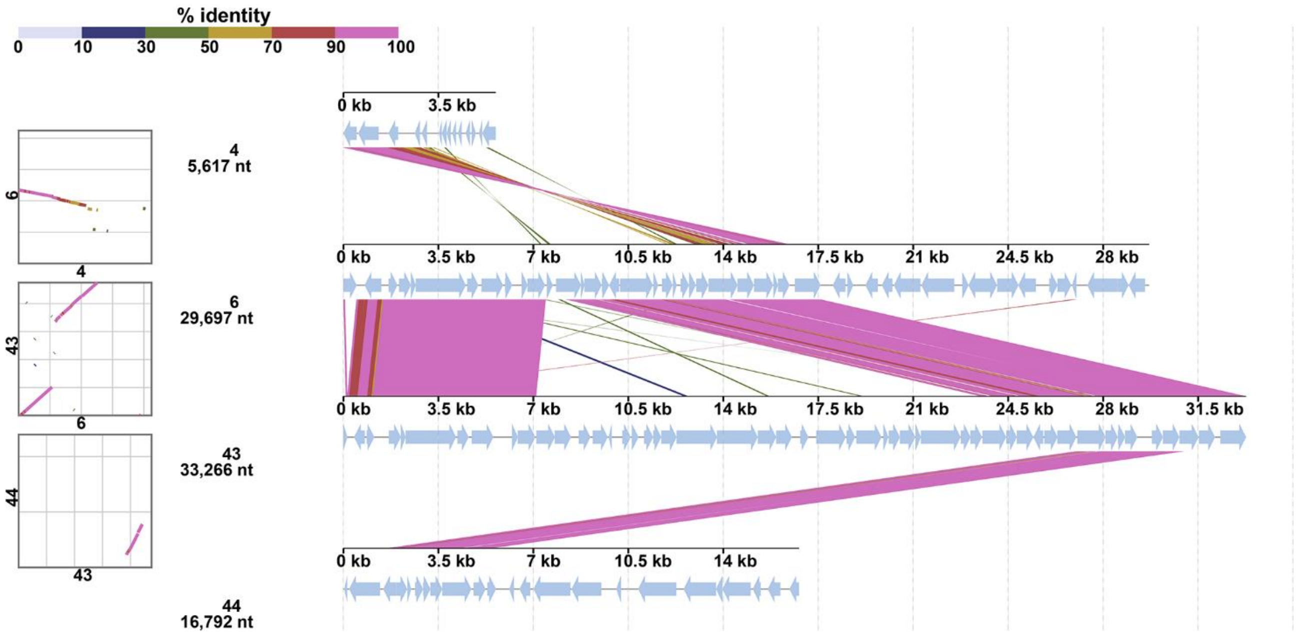

Figure S7. Alignment of prophage fragments in the *Sclerolinum annulatum* endosymbiont HMS1 genome, related to Figure 1D. Analyses were performed by the ViPTree online server.

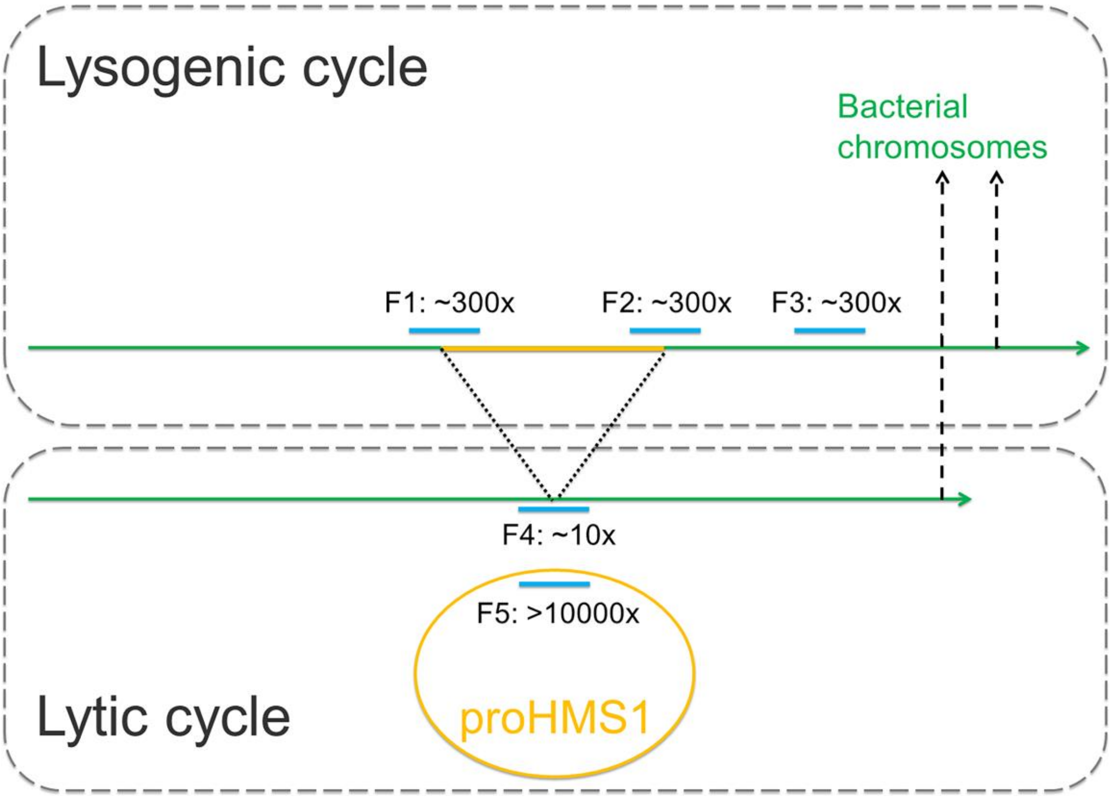

Figure S8. Schematic view of the Pacbio long reads mapping to the *Sclerolinum annulatum* endosymbiont HMS1 and prophage HMS1, related to Figure 1C and 1D. F1 to F5 are 1 kb fragments that match with different chromosome regions: F1 and F2 are overlapping regions of the lysogenic prophage and bacterial chromosome; F3 is a reference region on the bacterial chromosome; F4 is the bacterial region after prophage was released from the bacterial chromosome; F5 is a random region of the circular prophage proHMS1. Fragments of F1 to F5 were searched against Pacbio long reads with BLASTN and the matched reads were summarized as estimated coverage (x) of respective regions.

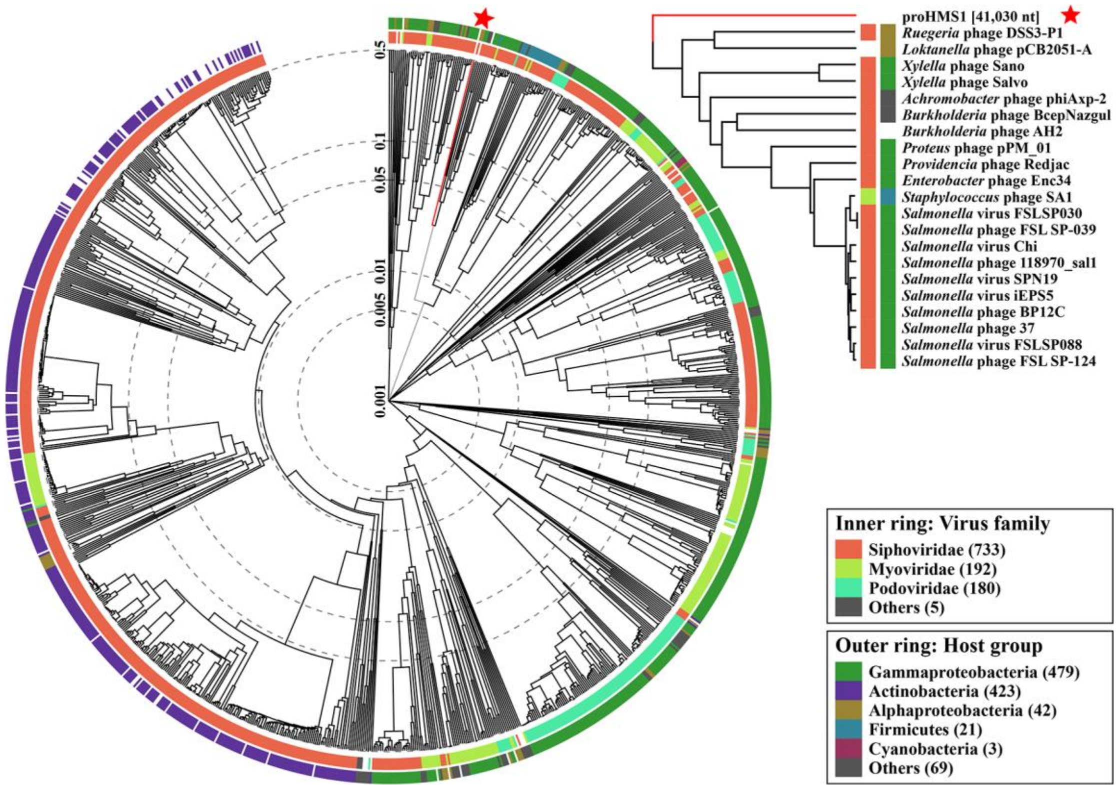

Figure S9. Proteomic tree of the prophage proHMS1 and its relatives using the ViPTree online server, related to Figure 1D.

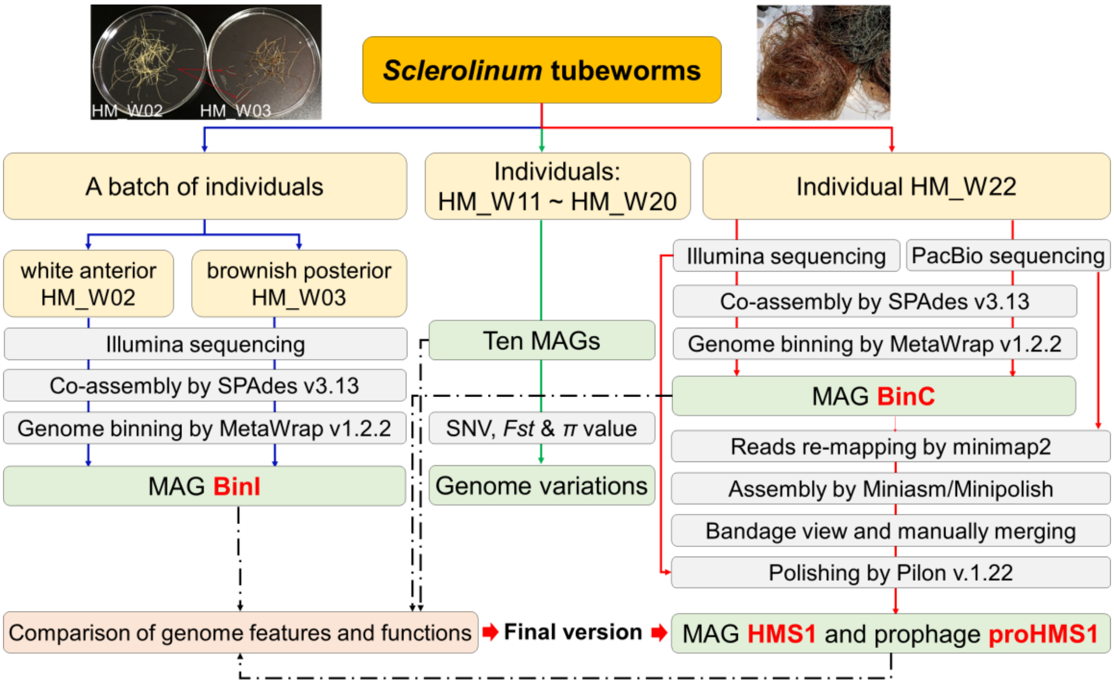

Figure S10. Schematic representation of the assembly strategy for the *Sclerolinum annulatum* endosymbiont and the phage proHMS1, related to STAR Methods. Abbreviation: MAG, metagenome-assembled genome.

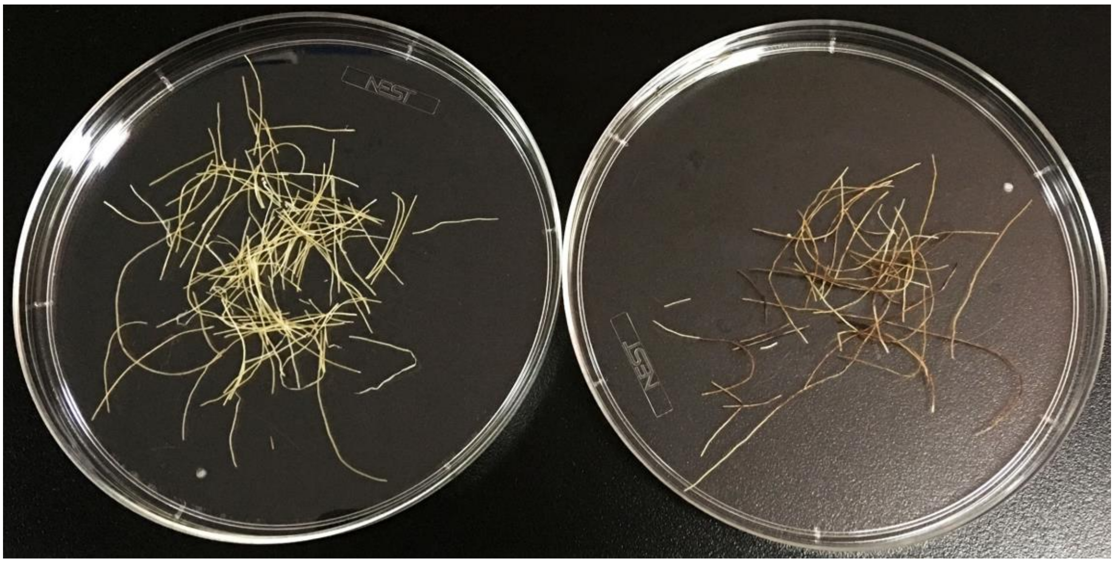

Figure S11. Different parts of the *Sclerolinum annulatum* specimens, related to STAR Methods. Tubeworm specimens were cut into two parts based on their colors after being rinsed with 100% ethanol, the pale white anterior parts (left, HM_W02) and the brownish posterior part (right, HM_W03), respectively.

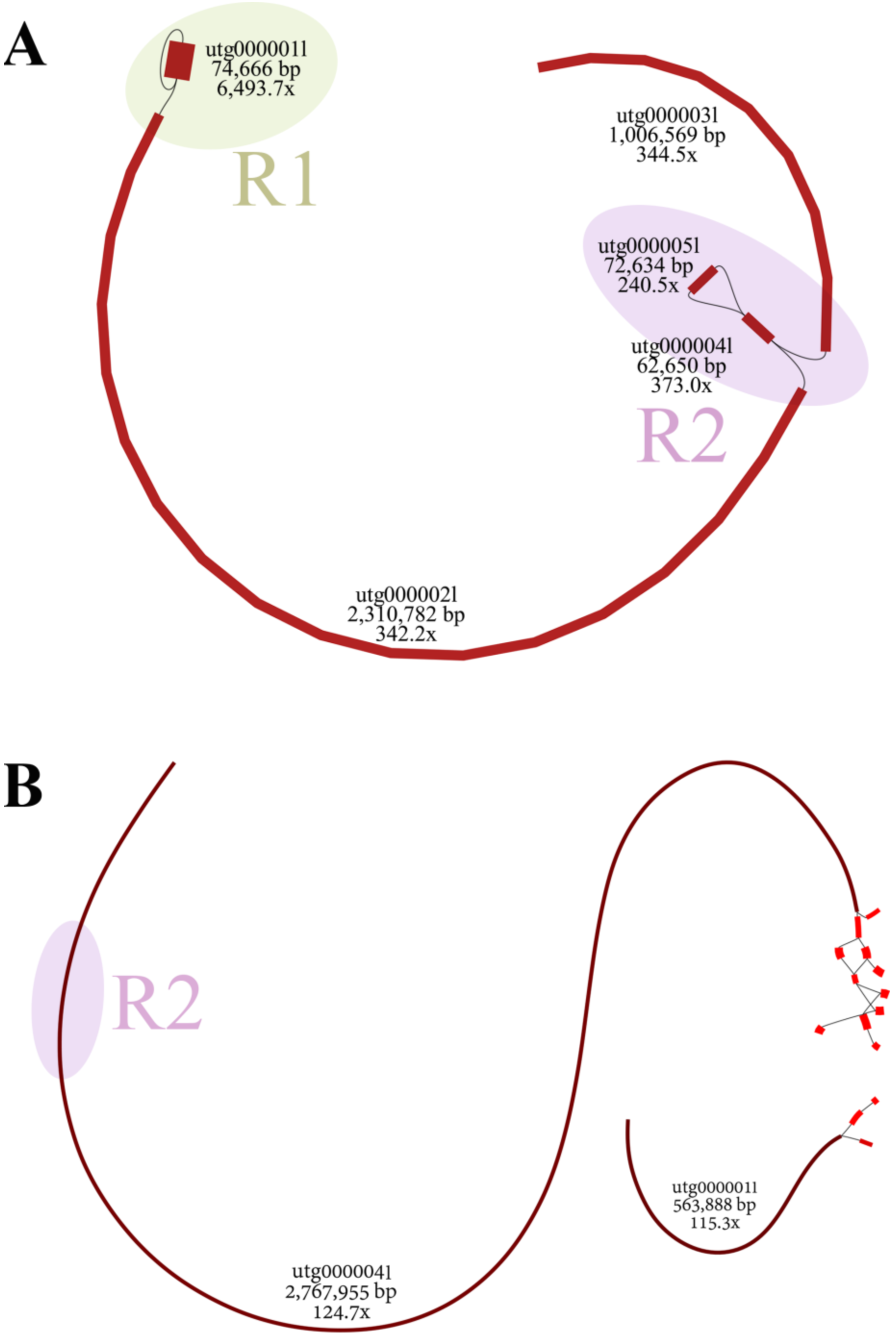

Figure S12. Bandage view of metagenomic nodes for the finished genome HMS1, related to STAR Methods. A Metagenomic nodes assembled from qualified PacBio long reads. **B** Metagenomic nodes assembled from raw PacBio long reads. Node length was correlated with the length of metagenome-assembled fragments and node width represents the depth of Illumina short reads on the fragments. Node length and width are relative values and they are not the same in the two figures.

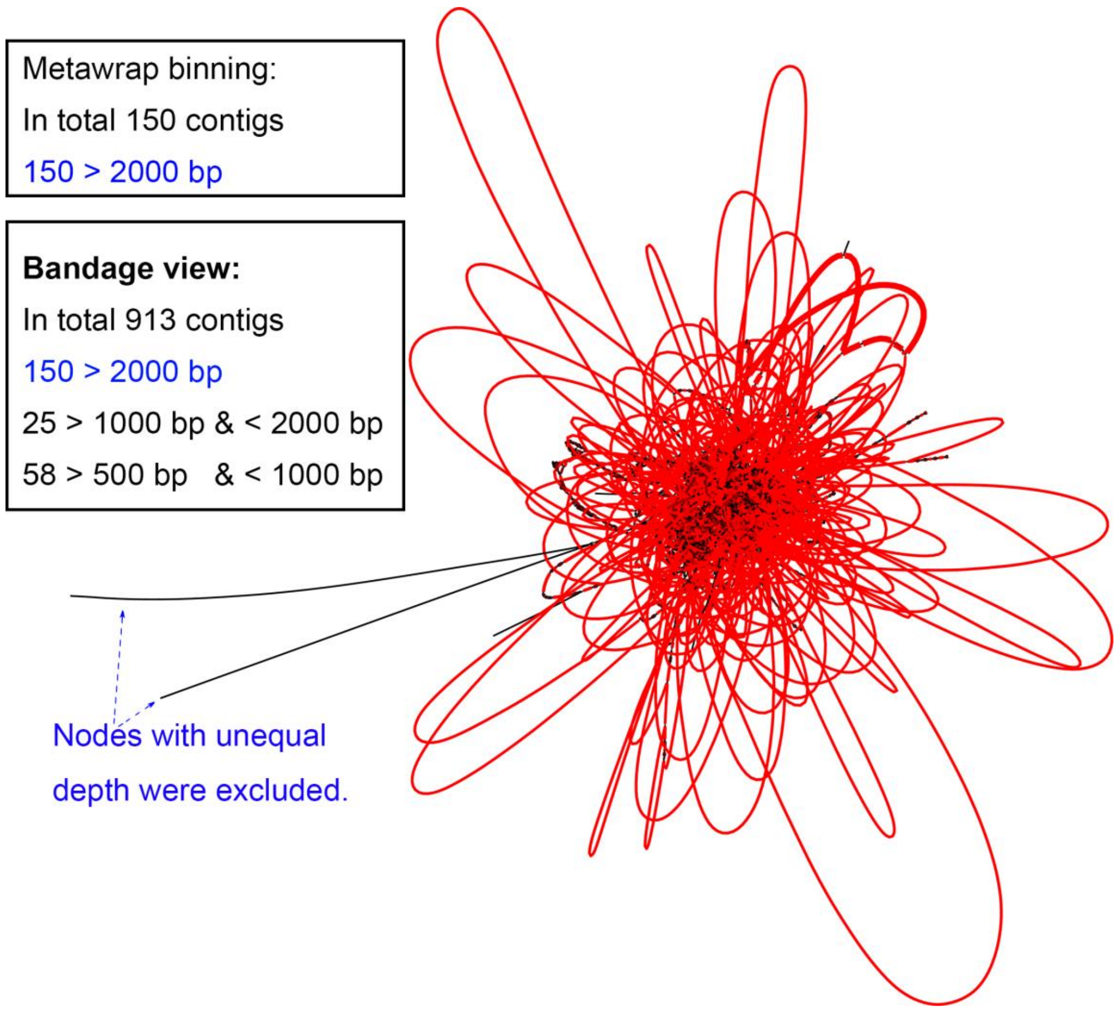

Figure S13. Bandage view of metagenomic nodes for the genome BinI, related to STAR Methods. Node length was correlated with the length of the metagenome-assembled fragments, and node width represents the depth of Illumina short reads on the fragments.

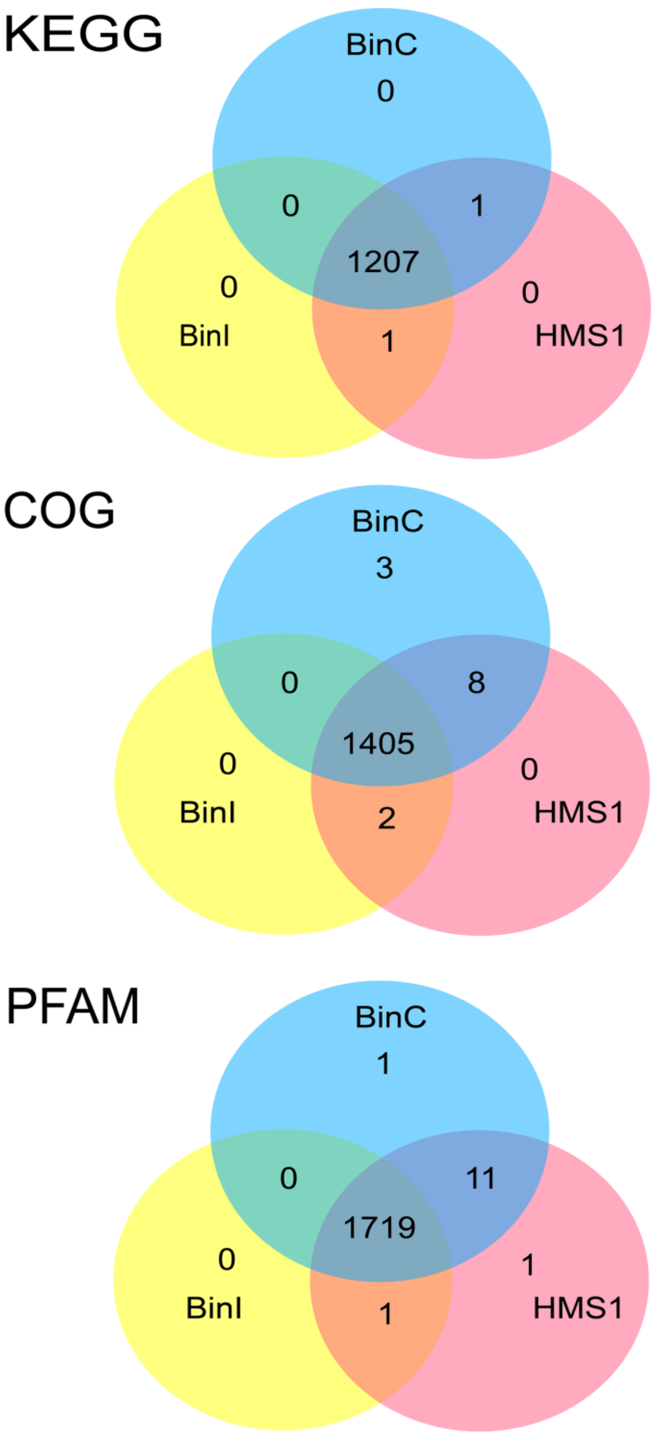

Figure S14. Venn diagrams showing the functional convergence of three genome bins, related to STAR Methods. Genomes HMS1, BinC, and BinI were assembled using different strategies and metagenome datasets, with details included in the Materials and Methods section. Functions were annotated by searching against the KEGG/COG/PFAM databases.
